# The *Arabidopsis* bZIP11 transcription factor links low-energy signalling to auxin-mediated control of primary root growth

**DOI:** 10.1371/journal.pgen.1006607

**Published:** 2017-02-03

**Authors:** Christoph Weiste, Lorenzo Pedrotti, Jebasingh Selvanayagam, Prathibha Muralidhara, Christian Fröschel, Ondřej Novák, Karin Ljung, Johannes Hanson, Wolfgang Dröge-Laser

**Affiliations:** 1 Julius-von-Sachs-Institut, Pharmazeutische Biologie, Julius-Maximilians-Universität Würzburg, Würzburg, Germany; 2 Department of Molecular Plant Physiology, Utrecht University, Utrecht, Netherlands; 3 Umeå Plant Science Centre, Department of Forest Genetics and Plant Physiology, Swedish University of Agricultural Sciences, Umeå, Sweden; 4 Umeå Plant Science Centre, Department of Plant Physiology, Umeå University, Umeå, Sweden; University of North Carolina, UNITED STATES

## Abstract

Plants have to tightly control their energy homeostasis to ensure survival and fitness under constantly changing environmental conditions. Thus, it is stringently required that energy-consuming stress-adaptation and growth-related processes are dynamically tuned according to the prevailing energy availability. The evolutionary conserved SUCROSE NON-FERMENTING1 RELATED KINASES1 (SnRK1) and the downstream group C/S_1_ basic leucine zipper (bZIP) transcription factors (TFs) are well-characterised central players in plants’ low-energy management. Nevertheless, mechanistic insights into plant growth control under energy deprived conditions remains largely elusive. In this work, we disclose the novel function of the low-energy activated group S_1_ bZIP11-related TFs as regulators of auxin-mediated primary root growth. Whereas transgenic gain-of-function approaches of these bZIPs interfere with the activity of the root apical meristem and result in root growth repression, root growth of loss-of-function plants show a pronounced insensitivity to low-energy conditions. Based on ensuing molecular and biochemical analyses, we propose a mechanistic model, in which bZIP11-related TFs gain control over the root meristem by directly activating *IAA3/SHY2* transcription. IAA3/SHY2 is a pivotal negative regulator of root growth, which has been demonstrated to efficiently repress transcription of major auxin transport facilitators of the *PIN-FORMED* (*PIN*) gene family, thereby restricting polar auxin transport to the root tip and in consequence auxin-driven primary root growth. Taken together, our results disclose the central low-energy activated SnRK1-C/S_1_-bZIP signalling module as gateway to integrate information on the plant’s energy status into root meristem control, thereby balancing plant growth and cellular energy resources.

## Author summary

Being in competition for reproductive success, plants use most of their photosynthetically produced energy resources to promote growth. However, under unfavourable environmental conditions plants also need to finance adaptive responses to ensure their survival. For this purpose a growth regulatory system is required to dynamically tune plant growth according to the plants’ prevailing energy status. Here, we characterize crucial components of this system that link plants’ energy management with root growth control. In detail, we demonstrate that a highly homologous group of energy-controlled regulators of the basic leucine zipper (bZIP) transcription factor family redundantly operate under energy deprivation to control expression of a determinant of hormonally-controlled meristematic root growth. By these means these regulators constitute a central hub to integrate detrimental environmental stress conditions, which converge on energy limitation, into plant growth. Understanding the interplay between the plants’ energy homeostasis and growth control are of major importance for future strategies to engineer efficient crop plants.

## Introduction

Sustaining energy homeostasis is of crucial importance for all living organisms to ensure their fitness and survival. In this respect, especially plants that cannot evade ever-changing environmental conditions due to their sessile lifestyle need to balance their input into highly energy-demanding growth processes according to the prevailing energy supply [1, 2]. For this reason, plants possess an energy management system, which induces catabolic processes and represses anabolism and plant growth under energy-deprived conditions [3, 4]. Central regulators are the evolutionary conserved, low-energy activated SUCROSE NON-FERMENTING1 RELATED KINASES 1 (SnRK1α1 (At3g01090), SnRK1α2 (At3g29160)), which accomplish massive transcriptional and metabolic reprogramming under low-energy stress [3, 5, 6]. Part of the SnRK1 response has been proposed to be exerted by basic leucine zipper (bZIP) transcription factors (TFs) of group S_1_ (bZIP1 (At5g49450), -2 (At2g18160), -11 (At4g34590), -44 (At1g75390), -53 (At3g62420)) and group C (bZIP9 (At5g24800), -10 (At4g02640), -25 (At3g54620), -63 (At5g28770)) [3, 7, 8]. Due to preferential heterodimerization, these bZIPs constitute the functionally interlinked C/S_1_ heterodimerization network [9–13]. Recently, SnRK1-mediated *in vivo* phosphorylation of the group C member bZIP63 has been demonstrated to enhance dimerization with the group S_1_ member bZIP11 and positions SnRK1 directly upstream of the C/S_1_ bZIP network [8]. Besides post-translational regulation by the low-energy responsive SnRK1 kinases, transcription of several C and S_1_ *bZIP* genes has been found to be energy-controlled. In particular, expression of group S_1_ bZIP1 and -53 as well as that of group C bZIP9, -25 and -63 is strongly induced by energy deprivation [8, 10, 14]. Moreover, translation of all group S_1_ members, including the highly homologous bZIP2, -11 and -44, is negatively regulated by SIRT (Sucrose Induced Repression of Translation) [15]. In this context, it was demonstrated that translation of S_1_ bZIPs is controlled by an evolutionary conserved upstream ORF (uORF), which encodes for a small sucrose control peptide, inhibiting main ORF translation under high sucrose levels [16–18]. In line with their proposed function in low-energy signalling, S_1_ bZIP translation was found to be strongly de-repressed under energy deprived conditions [19]. Although functional analyses of the SnRK1/bZIP pathway have frequently been performed under pronounced starvation conditions procured by extended night treatment [3], the system has also been found to operate in response to naturally occurring stress situations [7] or progressive energy depletion mediated by low-light cultivation [4].

The C/S_1_ bZIP network has been implicated in orchestrating low-energy triggered catabolism [10, 20]. This includes, in particular, starvation induced breakdown of branched-chain amino acids to fuel primary metabolism [10]. In addition to their metabolic impact it has been demonstrated that specifically the highly homologous bZIP11-related TFs (bZIP2, -11, -44) quantitatively modulate auxin-responsive gene expression by recruiting the histone acetylation (HAT) machinery to target promoters [21]. In this respect, especially members of the *Aux/IAA* (*AUXIN/INDOLE-3-ACETIC ACID*) and *GH3* (*GRETCHEN HAGEN 3*) gene families, which present crucial negative feedback regulators of auxin signalling and homeostasis, were found to be addressed.

Auxin signalling is well-known to play a key role in coordinating root growth and development [22]. Therefore, spatio-temporally controlled auxin transport is required to maintain and promote meristematic activity at the root apical meristem (RAM) or beneath the prospective sites of root organ formation [23]. By modulating expression of auxin transport facilitators of the PIN-FORMED (PIN) protein family, such as that of PIN1 (At1g73590) and PIN3 (At1g70940) [24–26], the phytohormones cytokinin and auxin antagonistically control polar auxin transport (PAT), thereby determining meristem size and consequently growth rates of the primary root [27]. Several preceding studies revealed that this dynamic process is mechanistically accomplished by a key intersection between both hormonal pathways, constituted by the cytokinin-responsive and auxin-labile INDOLE-3-ACETIC ACID INDUCIBLE 3 / SHORT HYPOCOTYL 2 (IAA3/SHY2) (At1g04240) repressor. Importantly, it could be demonstrated that this pivotal regulator directly represses *PIN* transcription and thereby controls PAT, RAM activity and primary root growth [28–30]. Recently, *IAA3/SHY2* expression has been found to be controlled by bZIP11 via its HAT recruitment mechanism [21], suggesting that bZIP11 and closely related TFs constitute a gateway to integrate low-energy related stimuli into auxin-mediated root growth responses [21, 31–33].

In fact, root growth is well-known to show a high phenotypic plasticity in response to energy-demanding stress situations [1]. In particular, the primary root has been found to react rapidly to progressive energy depletion by arresting its growth [3, 34, 35]. The molecular players involved in this crucial adaptive response remain, however, unknown. In this study, we disclose the pivotal role of the low-energy activated bZIP11-related TFs as negative regulators of primary root growth under starvation conditions.

We propose that via controlling the starvation triggered expression of *IAA3/SHY2* - a central component of root meristematic activity—the discussed bZIPs repress *PIN* expression and thereby interfere with PAT and, consequently, auxin-driven root growth. By this elegant mechanism bZIP11-related TFs provide means to adjust primary root growth in accordance to diverse detrimental environmental stimuli that converge on intracellular energy limitation.

## Results

### Starvation triggered *IAA3/SHY2* transcription depends on bZIP11-related TFs

Previous reports clearly demonstrated the impact of IAA3/SHY2 in restricting auxin driven meristematic root growth [28–30, 36]. As expression of this important root growth regulator was found to be triggered by starvation stimuli [33] and by the highly homologous, low-energy activated bZIP11-related TFs (bZIP2, -11, and -44) [21], we hypothesised that these bZIPs might interfere with primary root growth by inducing *IAA3/SHY2* expression under energy deprivation. In order to address this question, we aimed at monitoring *IAA3/SHY2* transcript abundance in *bZIP* mutant plants. As unfortunately (I) no T-DNA insertion mutants were available for *bZIP11* or *bZIP2*, (II) recent studies disclosed functional redundancy in activating *IAA3/SHY2* transcription by all three related bZIPs [21] and (III) individual bZIP knockdown approaches applying artificial microRNA (amiRNA) based techniques [37] were hampered by the high sequence homology between *bZIP11* and its closely related factors, we generated an Estradiol (Est)-inducible amiRNA-bZIP2/-11/-44 line (XVE-ami2/11/44) utilizing the well-established XVE-system [38]. This approach enabled both, simultaneous transcript reduction of all three bZIPs and due to its inducibility the analysis of direct TF controlled responses. Moreover, it was straight-forward to circumvent putative lethality of a triple null-mutant. The efficiency and specificity of the transgenic knock-down line was determined *in planta* by quantitative real-time PCR (q-RT-PCR). By these means an Est-mediated reduction of the corresponding *bZIP* (*bZIP2*, *-11* and *-44*) transcripts to roughly 20 to 40% [21] and not of closely related ones, such as those of *bZIP1* or *bZIP53* could be detected (S1 Fig).

**Fig 1:**
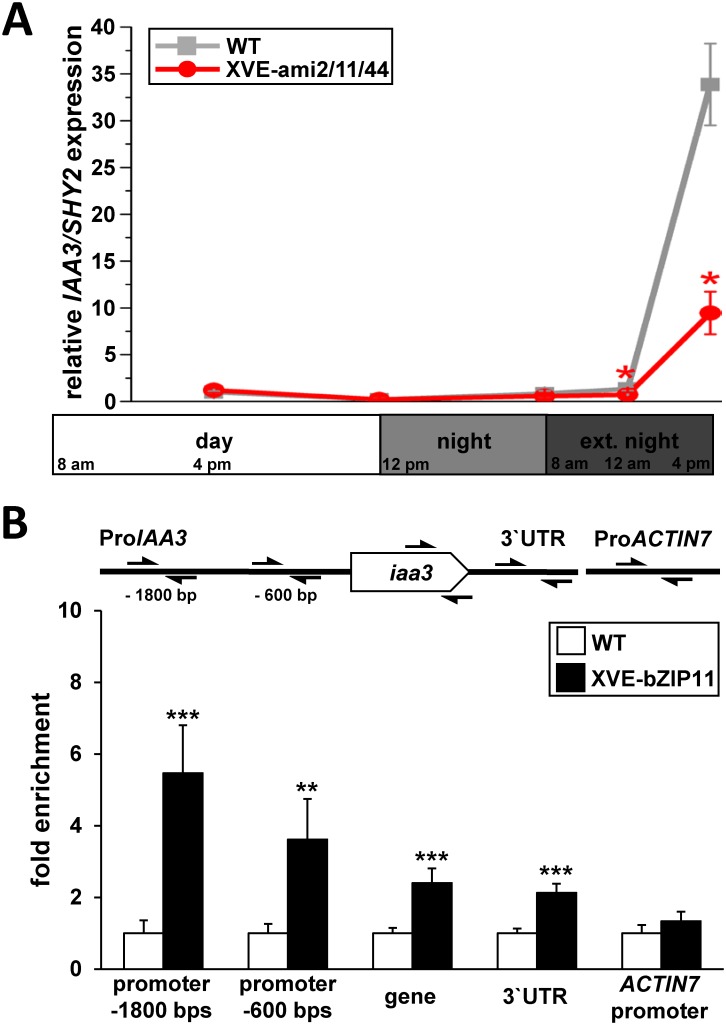
bZIP11 directly binds the *IAA3/SHY2* promoter and is crucial for its starvation induced transcription. **A**) Expression of *IAA3/SHY2* was quantified in Est-treated WT (grey line) and XVE-amiRNA-bZIP2/11/44 (line 2) plants (red line) applying q-RT-PCR. 2-weeks old, aseptically grown plants were treated with 10 μM Est at the beginning of the day and root material was harvested at distinct time-points of the day (middle and end of day), night (end of night) and extended night (4 and 8 h). Presented are mean expression values from 3 independent plant pools per genotype (+/- SEM), relative to *UBQ5* expression and normalized to WT at the middle of day (set to 1). Significant differences between genotypes for each individual time-point were determined by Student’s *t*-test and are labelled with asterisks (* p ˂ 0.05). **B**) Direct binding of HA-tagged bZIP11 to *IAA3* promoter, coding or 3’ UTR sequences as well as to the *ACTIN7* promoter (as indicated by arrows) was assessed by ChIP. Root material of XVE-bZIP11 plants was harvested at the middle of day after 8 h of Est treatment (10 μM). Given is the mean abundance (+/- SEM), (n = 4) of precipitated DNA-fragments in induced XVE-bZIP11 plants (black bars) relative to WT (white bars) as determined by q-RT-PCR. Significant differences between genotypes for each amplified DNA region were defined by Student`s *t*-Test and are labelled by asterisks (** p ˂ 0.01; *** p ˂ 0.001).

Making use of the knock-down and respective WT plants, we analysed *IAA3/SHY2* transcript abundance in presence of Est, in roots at defined time-points of the day, night and extended night applying q-RT-PCR (Fig 1A). Although no significant differences in *IAA3/SHY2* expression were observed between the genotypes under a 16 h day / 8 h night growth regime (long day, LD), strong bZIP dependency became apparent under energy deprived conditions provoked by short-term (4 or 8 h) extended night treatment. In fact, *IAA3/SHY2* expression, which continuously increased with duration of extended darkness in WT, was significantly alleviated in the multiple bZIP2/11/44 knock-down line. These data suggest, that bZIP activity is crucial to transduce low energy rather than light- or clock-related signals into *IAA3* transcription. In order to address whether *IAA3* expression is directly controlled by bZIP11-related TFs, we performed ChIP (Chromatin immunoprecipitation) using root material of XVE-bZIP11 plants. After Est-mediated bZIP11 induction, we observed a strong enrichment of precipitated *IAA3* promoter fragments compared to the WT control (Fig 1B). In contrast, fragments corresponding to the *ACTIN7* (At5g09810) promoter or to *IAA3* coding and 3`UTR regions were only marginally enriched. The minor enrichment of coding and 3`UTR sequences was likely attributed to limited ChIP resolution of approximately 1000 bps. These findings, which are in line with previously published *in vitro* binding studies [39], suggest that bZIP11 is able to directly target the *IAA3* promoter, presumably by binding to a cognate G-box *cis*-element located around -1800 bps apart from the translational start site. An additional ChIP signaI identified in the -600 bps promoter region could be explained by binding to a G-box like (TACGTG) motif. These findings support the view that the bZIPs under investigation interfere with the IAA3 controlled root growth regulatory system under starvation conditions.

### bZIP11-related TFs control starvation triggered repression of root growth

**Fig 2:**
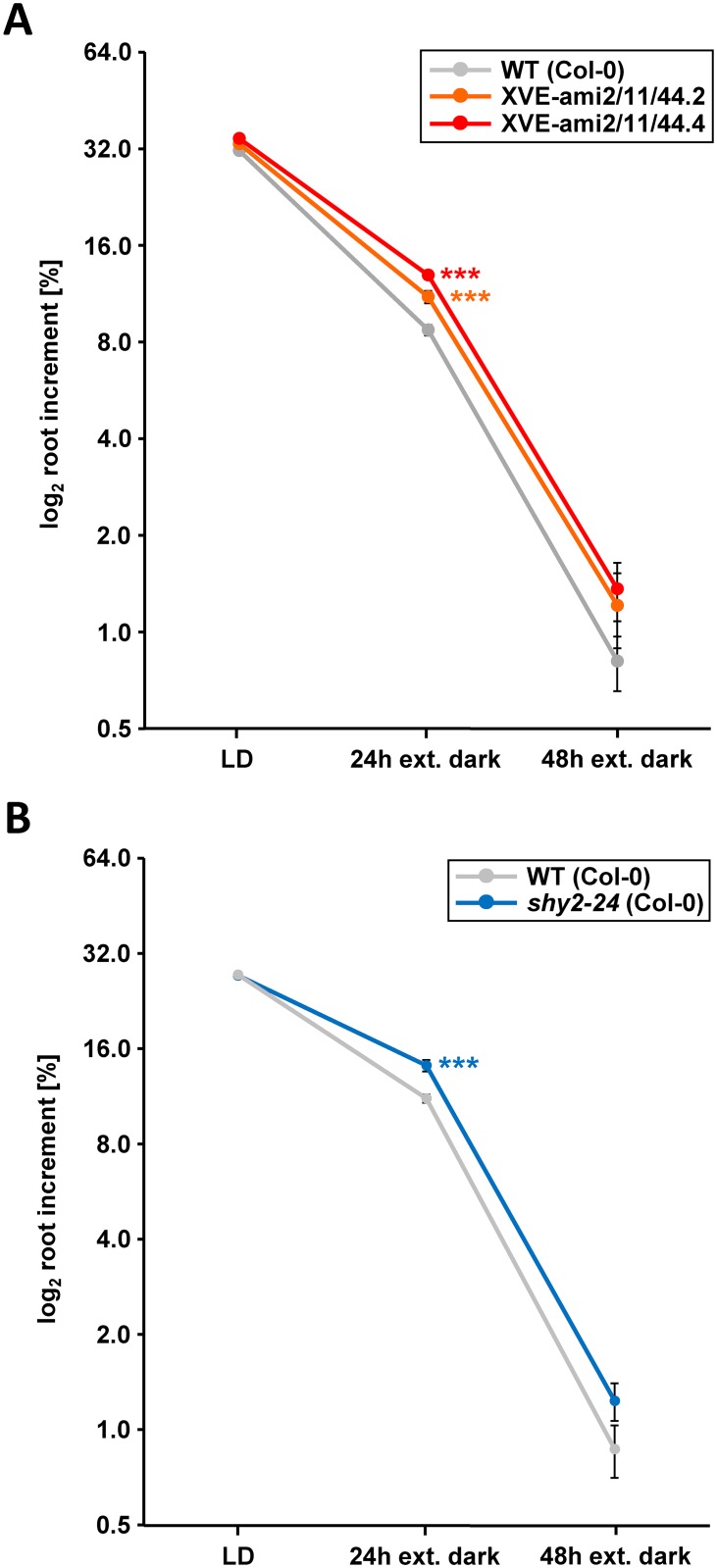
bZIP11-related TFs and IAA3/SHY2 control starvation-triggered repression of root growth. Root increment of 2-weeks old *Arabidopsis* WT and **A**) 2 independent aseptically grown XVE-ami2/11/44 lines (line 2 and 4) or **B**) *iaa3* loss-of-function plants (*shy2-24* in Col-0 background) was analysed under long day (LD) and extended night (24 and 48 h) conditions. **A**) 2 days before plants were cultivated under individual growth conditions they were transferred to MS medium supplemented with 10 μM Est. **A and B**) Presented is the mean log_2_ root increment (+/- SEM) of 50 individual plants per plant line and treatment. Significant differences between genotypes for each individual treatment were determined by Student’s *t*-test and are labelled with asterisks (*** p ˂ 0.001).

Progressive energy deprivation has been found to rapidly result in primary root growth repression, which could be countervailed by exogenous sugars [34]. As bZIP11-related activity was found to be negatively correlated with intracellular sugar levels [16, 17, 19] and required to induce expression of the negative root growth regulator IAA3/SHY2 under starvation conditions, we hypothesized that bZIP11-related factors might control root growth depending on the prevailing sugar availability. To test this assumption, we monitored primary root growth of WT and XVE-ami2/11/44 plants under energy-deprived and high-energy conditions. In fact, energy depletion induced by cultivating the plants on MS medium without sugars, but in presence of the photosynthesis inhibitor DCMU (3-(3,4-dichlorophenyl)-1,1-dimethylurea) resulted in significantly reduced root growth, which could be by-passed by feeding external glucose (S2A Fig). Strikingly, this starvation-triggered root growth repression was much less pronounced in XVE-ami2/11/44 plants compared to WT, demonstrating that the knock-down plants failed to respond appropriately to energy deprivation by reducing root growth. These results could be confirmed using extended darkness as an alternative low-energy condition. Again, root growth was significantly less repressed in two independent XVE-ami2/11/44 knock-down lines compared to WT after 24 hours of extended night (Fig 2A and S2B Fig). However, it has to be noted that after 48 h of prolonged darkness both genotypes comparably showed severe root growth inhibition, which was likely caused by substantial energy exhaustion. Importantly, considering both alternative low-energy approaches, it has to be noted that no differences in primary root growth could be observed between WT and bZIP knock-down plants when cultivated in the presence of exogenous sucrose (S2A Fig) or under LD growth conditions (Fig 2A and S3B Fig). Finally, we also examined the response of *iaa3* loss-of-function plants (*shy2-24*) to energy deprivation provoked by extended darkness (Fig 2B and S2C Fig). As *iaa3* mutants revealed a comparably low responsiveness to the low energy situation as the bZIP knock-down lines we concluded that the respective bZIPs largely operate via the crucial root growth regulator IAA3/SHY2 to adjust root growth in accordance to energy availability. However, we cannot rule out at this point that other bZIP targets, particularly other Aux/IAAs might also contribute to a minor extend.

### bZIP11-related TFs interfere with auxin-mediated root growth

**Fig 3:**
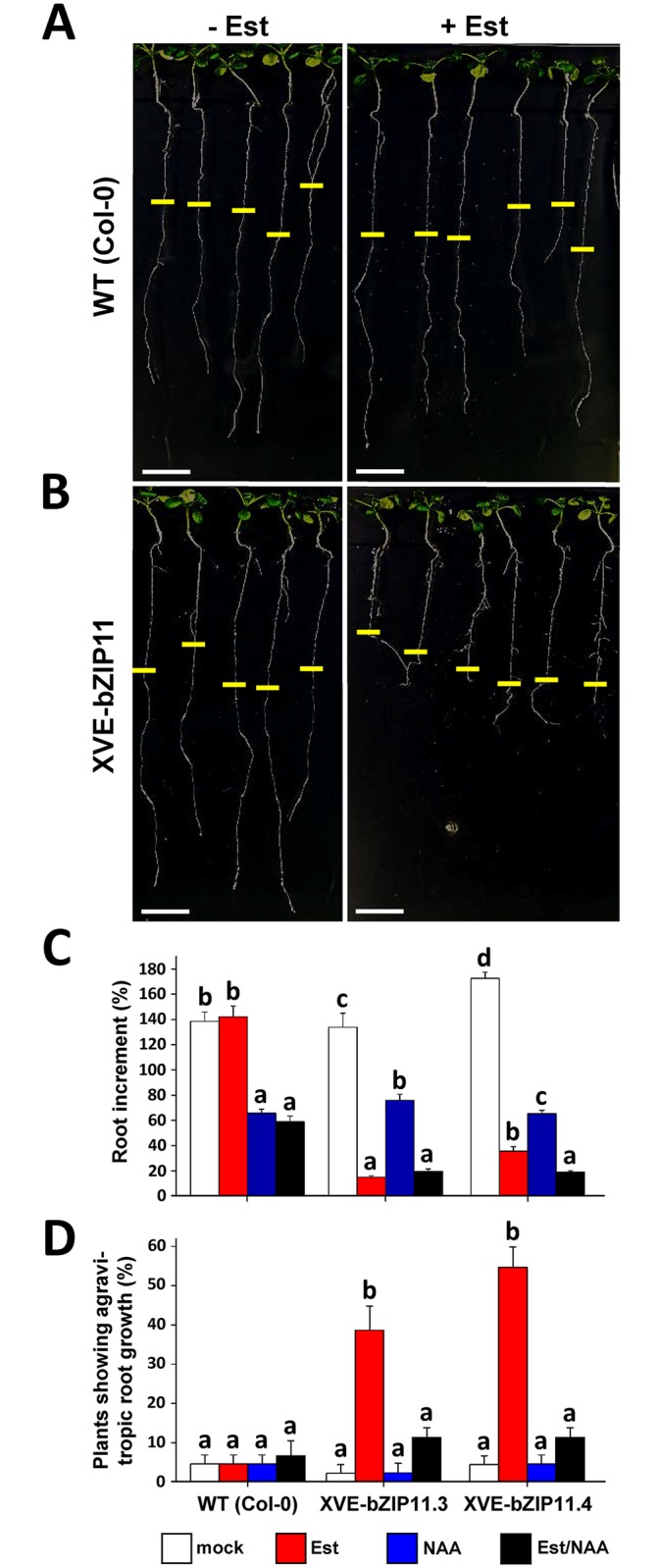
bZIP11 expression impairs auxin-related root growth responses. Auxin-related root growth responses in WT and XVE-bZIP11 (line 3 and line 4). Plants were cultivated for 2 weeks on ½ MS plates without sugars under LD regime and subsequently transferred for another week on inductive medium supplemented with 10 μM Est (red bars), exogenous auxin (0.25 μM NAA, blue bars), a combined Est and NAA treatment (black bars) or DMSO as solvent control (white bars). Representative pictures of (**A**) WT (Col-0) and (**B**) XVE-bZIP11 (line 3) in presence or absence of the Est inducer, respectively. Yellow bars mark root length before inductive treatments. Scale bar = 1 cm. Root parameters such as (**C**) increment of the primary root and (**D**) plants showing agravitropic root growth were quantified from 40 individual plants per treatment and genotype. Presented is (**C**) the mean percentage of root increment relative to root length before treatment or (**D**) mean percentage of plants showing agravitropic roots growth (+/- SEM). Significant differences between the treatments for each individual genotype were determined by one-way ANOVA and Tukey post-hoc test and are labelled with different letters.

Expression of bZIP11, as well as that of its close homologs bZIP2 and bZIP44 is well-characterised to be repressed by sucrose [16, 17] and induced by low energy conditions [19]. In order to specifically analyse their mechanistic impact on root growth independent from the dark/starvation stimulus, which might affect several physiological plant responses, we made use of transgenic gain-of-function approaches of these bZIPs. Constitutive expression of *bZIP11* [20] or its target *IAA3/SHY2* [33] has been found to result in severe shoot and root growth repression. Hence, we generated Est-inducible over-expressers of HA-tagged bZIPs, enabling spatio-temporally controlled transgene expression. By this means, stable transgenic lines of *bZIP2* (XVE-bZIP2.2), *bZIP11* (XVE-bZIP11.3 and XVE-bZIP11.4) and *bZIP44* (XVE-bZIP44.3 and XVE-bZIP44.9) were obtained, which showed an inducible moderate transgene expression as confirmed by Western Blot analysis (S3A Fig). To monitor root architecture of wild-type (WT) and transgenic XVE lines in presence and absence of Est, we cultivated 2-weeks old plants from each line for 1 week under LD regime on MS-medium supplemented with inducer or solvent, respectively. Whereas the WT did not show any noticeable response to Est treatment (Fig 3A, 3C and 3D), the XVE-bZIP2, -11 and -44 lines exhibited several low-auxin phenotypes at the most distal root part, such as strongly reduced primary root growth (Fig 3A–3C and S3B Fig), impaired auxin-induced root hair formation (S1 Table) and agravitropic root growth (Fig 3A, 3B, 3D and S3C Fig).

As these phenotypes are indicative of local auxin depletion at the root apex, we tested whether co-application of low to moderate levels of exogenous auxin (0.001 to 1 μM 1-naphthaleneacetic acid, NAA) were able to at least partially revoke the bZIP-associated root responses. In fact, we observed that application of low levels of NAA ranging from 0.01 to 0.1 μM antagonized bZIP-mediated primary root growth repression (S3D Fig), although they were shown to act (as a consequence of too high inner cellular auxin levels) slightly inhibitory on WT root growth [40]. More strikingly, root gravitropism could be largely rescued by moderate NAA concentrations (0.1 to 0.25 μM) (Fig 3D and S3E Fig). Individual or joint application of lower (0.001 μM) or higher NAA concentrations (up to 1 μM) had no significant effect or resulted in strong root growth inhibition, respectively (S3D and S3E Fig). In sum, these results strongly suggest that bZIP-mediated root growth inhibition is accomplished by repression of auxin signalling within and/or impairment of basipetal auxin transport to the distal meristematic root zone.

### Root-localized *PIN1* and *PIN3* are repressed by bZIP11

**Fig 4:**
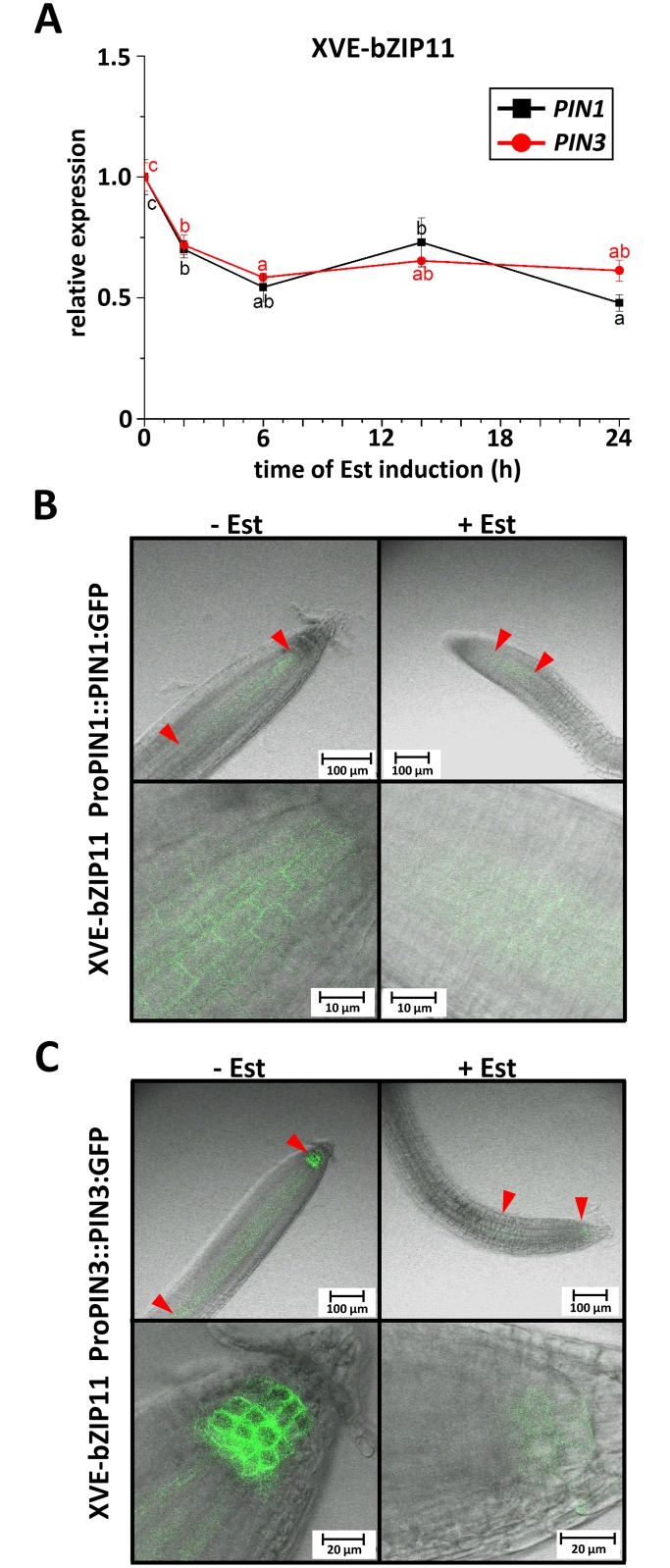
Root *PIN1* and *PIN3* expression is repressed by bZIP11. **A**) Transcript abundance of *PIN1* and *PIN3* were determined in XVE-bZIP11 plants (line 4) up to 24 h after Est induction using q-RT-PCR. Presented are mean *PIN1* (black line) and *PIN3* (red line) expression levels from 3 individual plant pools per line and time-point (+/- SEM) relative to *UBQ5* transcription and normalized to un-induced conditions (set to 1). Significant differences between induced and un-induced conditions for each gene were determined by one-way ANOVA followed by Tukey post-hoc test and are marked by individual letters. **B**) PIN1 and (**C**) PIN3 protein abundance was assessed by confocal laser scanning microscopy in Est-induced (10 μM for 16 h) and un-induced XVE-bZIP11 plants (line 4), which were crossed with (**B**) ProPIN1::PIN1:GFP or (**C**) ProPIN3::PIN3:GFP reporter lines, respectively. Given are representative pictures from 10 individual plants per treatment and line. Red arrows indicate outlines of PIN expression domains. **B and C**) Scale bars represent 100 μm for whole root pictures (upper panels) and (**B**) 10 μm or (**C**) 20 μm for enlarged root tip images (lower panels). Quantifications are given in S4B Fig.

Members of the PIN gene family of auxin efflux carriers, such as PIN1, PIN2 (At5g57090), PIN3, PIN4 (At2g01420) and PIN7 (At1g23080), are well-known to implement cell-to-cell auxin transport in the root [25]. In particular, PIN1 and PIN3 were found to be the major auxin transport facilitators mediating polar auxin re-allocation from the shoot to the root tip [24, 25, 41]. As both *PIN1* [42] and *PIN3* (S4A Fig) have been shown to be repressed under energy deprived conditions and are known targets of the bZIP11-controlled IAA3/SHY2 repressor [32], we hypothesised that bZIP11 expression should result in reduced *PIN* transcription. In fact, this scenario would readily explain the low-auxin root growth phenotypes observed in bZIP over-expressors (Fig 3A–3D). Hence, we quantified *PIN1 and PIN3* expression in XVE-bZIP11 lines applying q-RT-PCR. Remarkably, Est-mediated *bZIP11* induction let to a rapid and strong repression of *PIN* transcription in roots (Fig 4A). In line with this observation, we found as early as 16 h after Est application a moderate to strong decline in root PIN1 and PIN3 protein abundance, as demonstrated by confocal laser scanning microscopy analysing XVE-bZIP11 plants, in a ProPIN1::PIN1:GFP or ProPIN3::PIN3:GFP background [43], respectively (Fig 4B and 4C and S4B Fig).

### Genuine *bZIP11* expression largely overlaps with PIN domains

*PIN1* and *PIN3* show locally defined expression patterns in roots. Whereas both are strongly expressed in the vascular bundle [44, 45], *PIN1* additionally shows high expression in endodermis cells of the root apex [46] and *PIN3* in root columella cells [24]. *IAA3/SHY2* expression has been found to largely resemble these PIN expression domains [30, 44, 45]. In order to examine if *bZIP11* domains overlap with that of *IAA3/SHY2* and *PINs*, we monitored genuine *bZIP* expression in the distal root. Therefore, *Arabidopsis* WT plants were stably transformed with a genomic *bZIP11* fragment, composed of the ~2300 bp promoter, the entire 5’ UTR leader, which was found to confer Sucrose Induced Repression of Translation (SIRT) [17, 18], followed by the *bZIP11* and *GFP* coding sequences.

**Fig 5:**
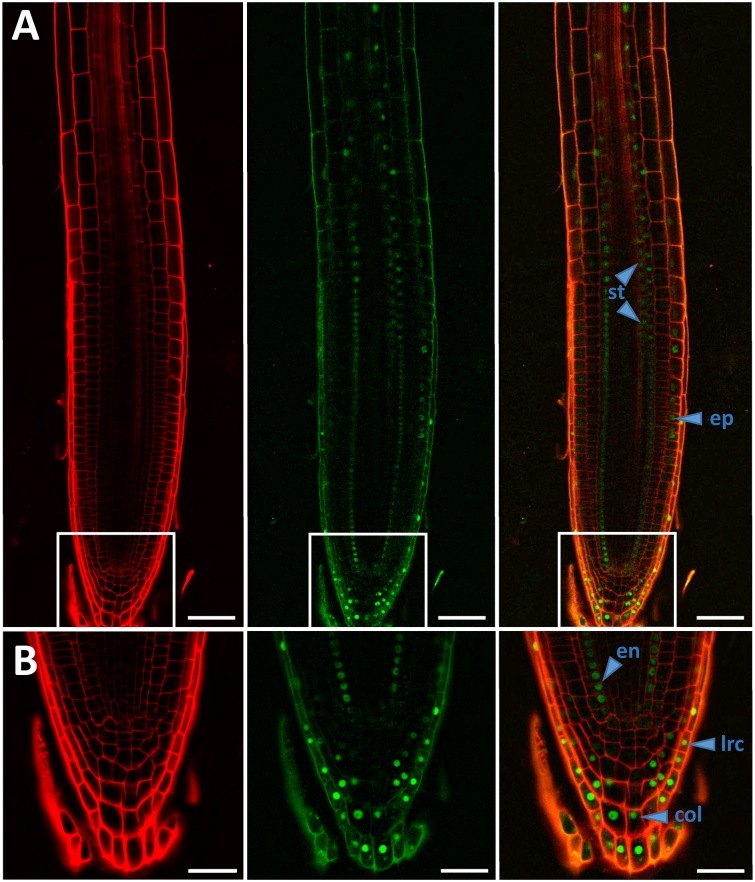
Genuine *bZIP11* expression in roots. **A and B**) Expression of *bZIP11* was analysed by confocal laser scanning-microscopy in roots of 10-days old *Arabidopsis* plants carrying a genomic *bZIP11* fragment (ProbZIP11_-2300bps_:5`UTR-bZIP11) fused to GFP. Plants were cultivated on ½ MS plates without sugars under a long day regime (16 h light / 8 h darkness) and analysed at the middle of the night period (4 h darkness). Given are representative pictures from 5 biological replicates. From left to right: outlines of root cells highlighted by propidium iodide, bZIP11-GFP fluorescence and merged pictures. **A**) Pictures depicting the whole meristematic root zone (scale bar: 40 μm). White insets mark regions, which have been enlarged and are presented (**B**) below the respective pictures (scale bar: 20 μm). bZIP11 expression domains in individual root tissues (ep: epidermis; en: endodermis; col: columella; lrc: lateral root cap; st: stele) are indicated by blue arrows.

Several reports demonstrated that bZIP11 functions in concert with the low-energy activated SnRK1 kinases in mediating metabolic reprogramming [3, 4, 7, 10]. Moreover its expression was found to be sucrose controlled [15, 16, 18, 19]. Hence, we analysed *bZIP11* expression under progressive energy depletion at the middle of the night applying confocal laser scanning microscopy (Fig 5A and 5B). Reproducibly, we observed a highly specific expression pattern, which was restricted to the lateral and columella root cap, the root epidermis from root tip up to the elongation zone and within the endodermal cell layer from root tip to the differentiation zone. Moreover, a weak signal could be detected in the root stele. As *bZIP* translation was shown to be promoted by pronounced energy starvation [19] and *IAA3/SHY2* as well as *PIN* expression was strongest in the central cylinder, we monitored stele-specific *bZIP11* translation efficiency under short-term (2 h) extended night conditions. Using the well-established Translating Ribosome Affinity Purification (TRAP) method [47], an increase in stele-specific *bZIP11* translation was observed under prolonged night when compared to expression at the middle of day (S5 Fig). In conclusion, genuine *bZIP11* expression domains observed under low-energy situations largely overlap with those described for *IAA3/SHY2* as well as that of *PIN1* and/or *PIN3*.

### bZIP11 impairs polar auxin transport to the root tip

**Fig 6:**
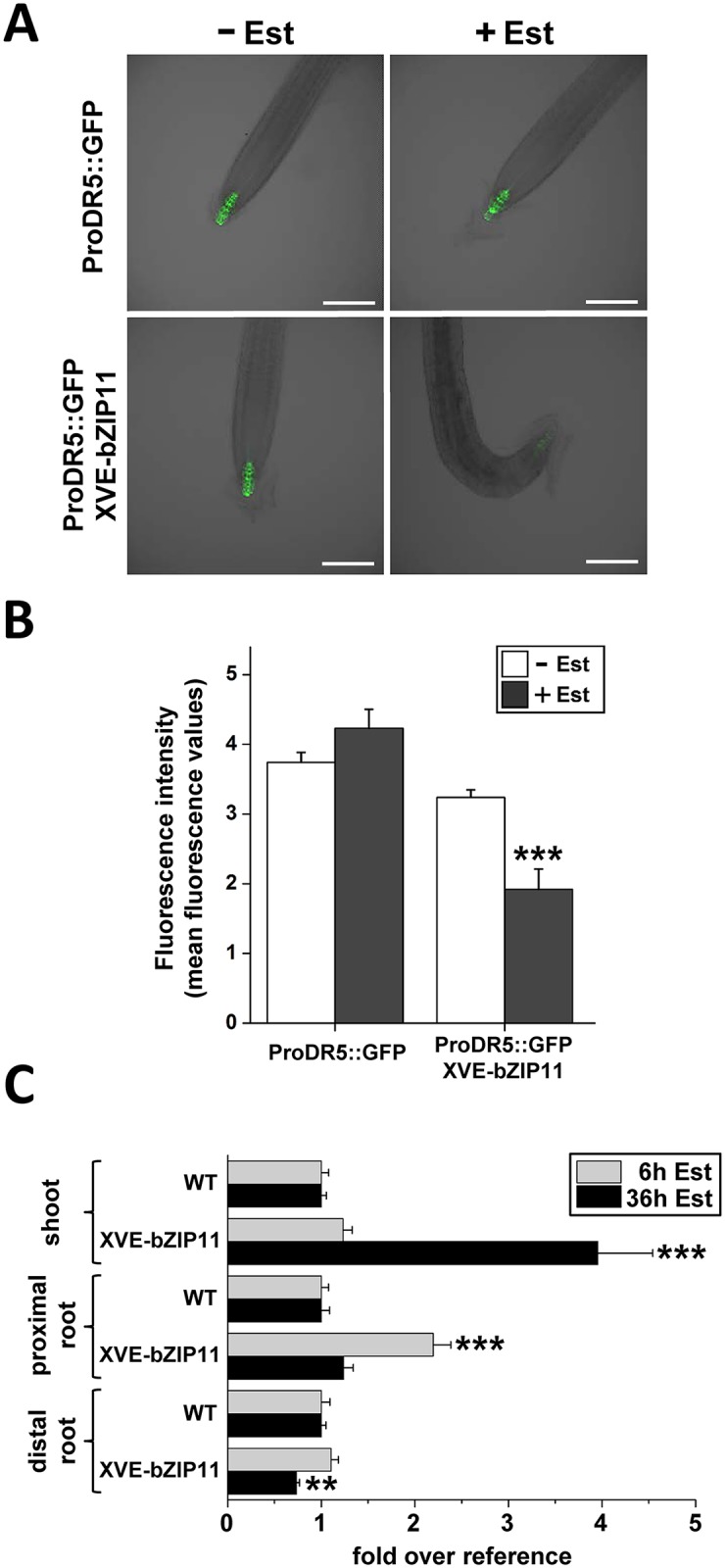
Root tip directed auxin transport is impaired by bZIP11. Root auxin signalling was analysed in WT and XVE-bZIP11 (line 4) by introducing an auxin-sensitive ProDR5::GFP reporter construct. **A**) Given are representative pictures. Scale bar = 150 μm. **B**) ProDR5 driven GFP fluorescence was quantified in WT and XVE-bZIP11 background from 40 individual plants per line and treatment in presence (grey bars) and absence (white bars) of Est (10 μM, 24 h) and is expressed as mean fluorescence values (+/- SEM). Significant differences between treatments were determined by Student’s *t*-test and are labelled with asterisks (*** p ˂ 0.001). **C**) Quantitative auxin measurements in the most distal root parts including the root tip, the proximal root including hypocotyl and the shoot of WT and XVE-bZIP11 plants suggest an impairment of auxin transport from the shoot to the root tip. Mean auxin levels (+/- SEM) were determined after 6 h (grey bars) and 36 h (black bars) of Est treatment from at least 5 pools of plants per line and treatment and are given relative to WT levels (set to 1). Significant differences between the genotypes for each time-point and root section were determined by Student’s *t*-test and are labelled with asterisks (** p ˂ 0.01; *** p ˂ 0.001).

IAA3/SHY2 mediated repression of *PIN* transcription has been found to result in impaired PAT from the shoot to the root tip [28]. As bZIP11, -2 or -44 induction was found to result in strong *IAA3/SHY2* expression [21] and to low-auxin phenotypes at the basal root part, we analysed whether bZIP11 might affect basipetal auxin transport processes. In order to get first insights into auxin transport, we monitored expression of an auxin response reporter, consisting of the synthetic DR5 promoter fused to GFP (ProDR5::GFP) [48, 49] in the root using confocal microscopy (Fig 6A). Whereas strong, root tip localised GFP fluorescence was Est-insensitive in the WT control, a significant Est-dependent decline could be observed in the XVE-bZIP11 line, suggesting again that basipetal auxin transport to or local auxin signalling within the root tip are impaired by *bZIP11* expression (Fig 6A and 6B). Similarly and consistent with bZIP11 function in low-energy signalling a short extension of the night period (8 hours) resulted in a strong decline in DR5-driven GFP expression in the root tip (S4A Fig).

In order to confirm the impact of bZIP11 on auxin transport and to characterize the bZIP11-mediated changes in auxin distribution, we quantitatively measured auxin concentrations in distinct segments of the plant. Applying liquid chromatography-tandem mass spectrometry (LC-MS/MS), we could indeed demonstrate that already 6 h after *bZIP11* induction a 2-fold increase of auxin compared to the WT control became apparent in the upper proximal root parts including the hypocotyl. 30 h later an even more pronounced shift of auxin to the shoot could be found (Fig 6C). This reduced auxin translocation from shoot to root, which rapidly let to significant auxin depletion in the most distal root parts can most likely be explained by bZIP-mediated impairment of PIN driven polar auxin transport (PAT) and is highly consistent with the observed bZIP-mediated low-auxin phenotypes at the root apex (Fig 3A–3D and S3B and S3C Fig and S1 Table). Similar results were obtained analysing XVE-bZIP44 plants (S6A–S6C Fig), supporting functional redundancy among the bZIP11-related factors.

### bZIP11 negatively affects root apical meristem size

**Fig 7:**
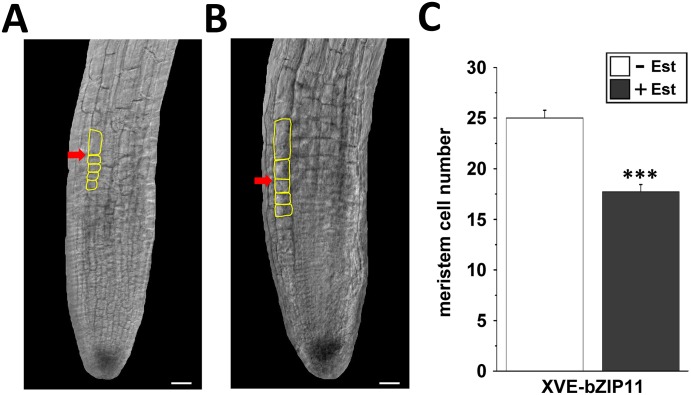
bZIP11 represses meristematic root growth. RAM size of XVE-bZIP11 plants (line 4) was assessed after 24 h of (**A**) solvent or (**B**) Est-treatment. Given are representative images. Yellow boxes highlight cortex cells in the TZ and a red arrow indicates the border between root meristem and root elongation zone. Scale bar = 20 μm. **C**) Mean meristem cell numbers (+/- SEM) from plants (n = 10) cultivated in the presence (grey bars) or absence (white bars) of Est. Significant differences between treatments were determined by Student’s *t*-test and are labelled with asterisks (*** p ˂ 0.001).

The antagonistic interplay between the phytohormones auxin and cytokinin is well-characterized to control root apical meristem size and consequently root growth rates [50, 51]. In particular, a high auxin to cytokinin ratio at the root tip is required to keep quiescent centre (QC) derived meristematic cells in their undifferentiated and rapidly dividing state. In contrast, decreasing auxin and increasing cytokinin levels in the more proximal root transition zone (TZ) drive cells to elongate and differentiate. By directly controlling PIN mediated basipetal auxin transport to the meristem, the bZIP11 target *IAA3/SHY2* has been found to determine root meristem size and hence root growth rates. In consequence, we analysed the impact of bZIP11 expression on meristematic root growth. Therefore, we assessed the RAM size by counting the file of cortex cells beginning from the QC to the first elongated cortex cell in the TZ [36]. By these means, we microscopically analysed the RAM of XVE-bZIP11 plants in the presence and absence of Est and found that *bZIP* expression let to a highly significant reduction of meristem size (Fig 7A–7C). Notably, this was also found to be true for the highly related bZIP44 TF (S7A–S7C Fig), again supporting functional redundancy.

## Discussion

### The energy-controlled bZIP11-related TFs act as rheostat to tune primary root growth in accordance to energy availability

Plants need to invest most of their resources into growth to ensure their reproductive success in highly competitive habitats. However, a significant proportion of their resources are generally consumed by adaptive responses to a broad range of environmental stresses. To balance the input of resources into these essential but conflictive processes of survival and fitness, a growth regulatory system is required to adjust growth according to the prevailing energy availability.

In fact, it is well-documented that plants are equipped with a sophisticated energy management system to react rapidly and sensitively to changes in the availability of carbon skeletons by adjusting their metabolism and growth [34, 35, 52]. The associated extensive re-programming was largely ascribed to the low-energy activated SnRK1s [3] and the counteracting sugar-responsive TARGET OF RAPAMYCIN (TOR) (At1g50030) kinases [35]. Whereas TOR has been found to play a profound role in anabolic processes and root growth promotion in the presence of sugars [35, 53], starvation triggered SnRK1 activity represses TOR signalling and drives plant catabolism and root growth repression [3, 54]. In accordance with their proposed function, plants constitutively expressing SnRK1s phenocopy the starvation-mediated repression of primary root growth [3, 34]. Notably, Est-inducible expression of *bZIP11* or the highly homologous *bZIP2* and *-44* TFs, which uncouples *bZIP* expression from repressive sugar regulation, results in similar primary root growth phenotypes. This suggests a partially redundant function among bZIP11-related factors and an interplay with SnRK1 kinases in energy-controlled primary root growth. A wealth of recent reports support this hypothesis showing that the group C/S_1_ bZIP heterodimerization network [11] exerts a significant proportion of SnRK1-mediated starvation responses [3, 4, 7, 8, 55]. In this respect, it has been highlighted that SnRK1s strongly enhance transcription mediated by bZIP11-related TFs [3] and promote bZIP11/bZIP63 heterodimerization by specific changes in the bZIP63 phosphorylation status [8]. Moreover, a significant overlap in global gene regulation provoked by moderate to severe energy depletion or expression of *SnRK1* or *bZIP11* has been observed [3, 4], indicating that expression of these regulators is sufficient to mimic natural starvation responses.

**Fig 8:**
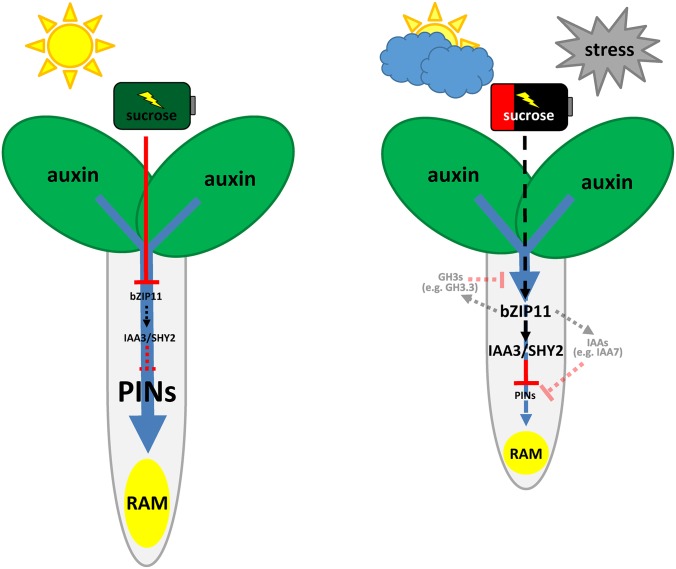
Proposed function of bZIP11 on auxin-mediated root growth in response to energy availability. Under optimal growth conditions plants utilize most of their photosynthetically produced sugars to support plant growth. To enable balanced plant shoot and root growth, shoot-synthesized auxin (blue arrow) is transported by PIN auxin efflux carriers to the RAM, resulting in meristem outgrowth and hence, elevated root growth rates. Whereas *bZIP11* translation is repressed under high sucrose levels, it is found to be strongly de-repressed under stress conditions, which converge on energy limitation. Induced by energy deprivation, bZIP11 directly controls transcription of the crucial root growth regulator *IAA3/SHY2*, which negatively affects *PIN* expression, leading to impaired polar auxin transport. Auxin depletion in the RAM promotes the differentiation of meristematic cells and represses root growth. By this means bZIP11 is able to dynamically control root growth in response to prevailing energy conditions. However, it cannot be excluded that other bZIP11 controlled *IAA* genes that might act redundantly with IAA3/SHY2 or *GH3s*, which are involved in auxin conjugation might also contribute to the observed root starvation response (indicated by faint, dashed lines).

In order to address the impact of bZIP11-related factors in energy-controlled primary root growth, loss-of-function studies were conducted. Considering putative functional redundancy among the highly homologous bZIP11-related TFs, Est-inducible knock-down plants were generated, in which an efficient and specific amiRNA-guided transcript depletion of all three bZIPs was simultaneously achieved [21]. Consistent with their proposed role in low-energy signalling, knock-down of the respective bZIPs strongly impaired the plant’s ability to respond to energy limitation by reducing root growth. Most importantly, no effects on root growth could be observed between WT and bZIP knock-down under photosynthesis supporting growth conditions or in presence of exogenous sugars, accentuating the impact of these regulators in low-energy triggered root growth control. Accordingly, starvation-induced root growth repression has been found to resume after transferring dark cultivated plants into light [42] or after exogenous glucose application [34], characterizing the energy-controlled regulatory circuit as reversible and dynamic. In line with this, bZIP11 translation has been found to be tuned by sugar availability, being de-repressed by energy deprivation [19] and repressed by glucose and sucrose [17, 18]. Moreover, transcription mediated by bZIP11-related TFs was found to be promoted by the low-energy sensing SnRK1s [3]. As bZIP11-related activity is thus manifold controlled by intracellular sugar levels that reflect the endogenous energy status, bZIP11-related factors are proposed to act as a hub in energy signalling, thereby providing means to reversibly tune root growth in response to stress situations which converge on energy limitation (Fig 8).

### Starvation triggered repression of meristematic root growth is controlled by bZIP11 and its target IAA3/SHY2

In our recent studies we demonstrated that bZIP11-related TFs modulate auxin responsive gene expression of a specific set of negative feedback regulators of auxin signalling and homeostasis [21, 56]. However, their impact on auxin-related phenotypes have not been addressed, yet. In this work gain-of-function approaches of the respective bZIPs revealed that their expression redundantly repressed primary root growth, which could be partially rescued by exogenous auxin, indicative of altered root auxin signalling and/or auxin transport processes being at work.

Furthermore, results obtained by analysing endogenous *PIN1* and *PIN3* expression in XVE-bZIP11 plants suggested that the bZIP-mediated low-energy response on auxin-controlled primary root growth is mechanistically accomplished by transcriptional repression of major auxin transport facilitators. In line with this, genuine *bZIP11* expression domains exhibited a significant overlap with that of *PIN1* and/or *PIN3* [24, 46]. However, as several reports highlighted bZIP11 as a strong activator of gene expression [20, 21], these data suggest that bZIP11-driven *PIN* repression is indirectly achieved. In fact, bioinformatics approaches on well-described auxin-responsive gene families emphasized the selective and conserved impact of bZIP-related *cis*-elements on promoters of *Aux/IAA* genes [31], which are potent negative regulators of auxin-controlled *PIN* expression [26]. In agreement with these results, we recently uncovered that bZIP11-related TFs quantitatively modulate expression of a specific subset of *Aux/IAAs* in *Arabidopsis* [21]. Importantly, expression of *Aux/IAA3* (*IAA3/SHY2*), which is a well-documented negative regulator of *PIN1* and *PIN3* expression in roots [28, 29, 36, 50], was found to be dependent on *bZIP11* expression and bZIP11-mediated recruitment of the histone acetylation machinery to its promoter [21]. Indeed, *bZIP11* mutants lacking the respective recruitment domain fail to activate *IAA3/SHY2* transcription [21]. Consistent with these results, we could confirm direct binding of bZIP11 to two G-box containing promoter regions, 1800 and 600 bps upstream of the *IAA3* start codon. Importantly, these binding regions are in line with a recently published cistrome data-set, analysing genome-wide TF binding sites *in vitro* [39]. Although promoter binding was detected under non-starved conditions to minimise competition for *cis*-elements by endogenous bZIP11 (Fig 1B), analyses on starvation stimulated binding would be of interest to disclose if additional post-translational mechanism operate to modulate bZIP11 binding specificity and strength as it has been previously proposed [8].

Remarkably, studies on IAA3/SHY2 demonstrated that expression of gain-of-function variants of this repressor largely resemble the bZIP11-induced root growth responses (primary root growth and root hair repression as well as agravitropism) [57] and disclose its decisive role in tuning meristematic root growth by modulating PIN1- and PIN3- mediated PAT [28, 30, 36, 50]. As low-energy controlled expression of *IAA3/SHY2* was found to mimic the bZIP11 expression profile - induced by starvation and repressed by sugars [33]- and was coherently found to be dependent on bZIP11-related TFs, *IAA3/SHY2* constitutes a well-suited link between the energy stimulus integrating bZIP11-related TFs and the plants’ basic root growth regulatory system.

Importantly, root growth studies employing *iaa3* and *bZIP* loss-of-function lines under energy deprived conditions, reveal a highly similar reduction in low-energy triggered repression of root growth for both genotypes. This suggests that bZIP11-related TFs largely exert their function on root growth via the root growth regulator IAA3. However, it has to be considered that TFs generally regulate several targets and hence further *bZIP11*-controlled mechanistic gateways might exist that interfere with RAM function. For instance, further starvation responsive genes that are involved in negatively regulating auxin signalling (e.g. *IAA7*) or auxin homeostasis (e.g. *GH3*.*3*) have been found to be bZIP11 controlled [21, 39].

Taken together, detailed genetic analyses and biochemical DNA binding studies propose a sequential activation of bZIP11, its target gene *IAA3/SHY2*, which in turn encodes the transcriptional repressor of *PIN* genes. As demonstrated in this study, this model is in line with the kinetic changes observed for transcripts and respective proteins of the involved players after Est-mediated bZIP induction. In fact, shortly after bZIP-mediated repression of *PIN* transcription (within 6 h), a successive reduction in PIN protein abundance (~ 16 h) as well as root tip auxin levels and primary root growth (~ 24 h) could be observed. Importantly, similar expression kinetics could be found under starvation conditions, supporting our assumption that the proposed signalling cascade is operating under physiological conditions. In this respect, it has been recently demonstrated that extended darkness rapidly activates bZIP11 via SnRK1-induced bZIP heterodimerisation [8]. Furthermore, we observed enhanced translation of *bZIP11* RNA in the stele after 2 h of energy limiting conditions (S5 Fig). These findings suggest an interplay between several post-transcriptional mechanisms acting to control bZIP11 function during starvation. Along this line, induced *IAA3/SHY2* transcription is observed within 4–8 h of extended night (Fig 1A), in which energy limitation increasingly becomes more severe. In accordance with enhanced expression of the IAA3/SHY2 repressor of *PIN* genes, a reduced level of PIN proteins should become visible after a short lag phase. Indeed, decreased PIN3-GFP levels were first observed after 8 h of extended night, steadily decreasing during the next 40 h. These dynamics of molecular events are in line with a decrease of free auxin in the root tip (S4A Fig) [58] and correlate with phenotypically reduced root growth 24 h after extended night (Fig 2A and 2B) [58], hence clearly supporting our working model.

Besides being expressed in roots, the *bZIP11* promoter has also been found to be active in the shoot apical meristem (SAM) and young leaves [18]. As dark-treatment also results in altered *PIN1* expression and auxin abundance in the SAM [59] as observed for the root meristem [42], it would be of great interest to analyse if bZIP11 mediates a more general starvation response on plant growth.

In summary, we disclosed in our work a crucial new aspect of bZIP11 function in plants’ energy management that is besides its well-documented impact on starvation triggered metabolic reprogramming, the control of meristematic root growth by modulating PAT. Hence, bZIP11 constitutes a central, energy-controlled hub, which provides means to integrate information on the plant’s energy status into plant metabolism and auxin-mediated growth responses. Understanding how plants balance growth in accordance to their energy supply is essential for developing future strategies to engineer plants for agricultural use.

## Materials and methods

### Plant material, transformation and culture

*Arabidopsis thaliana* Columbia (Col-0) XVE-bZIP2.2, XVE-bZIP11.3, XVE-bZIP44.3 and XVE-ami2/11/44.4 lines were generated using the “Floral Dip Transformation” technique [60] applying the *Agrobacterium tumefaciens* strain GV3101 and are characterized in this work (S1 and S3A Figs). XVE-bZIP11.4, XVE-bZIP44.9 and XVE-amibZIP2/11/44.2 plant lines have been described before [21]. For visualization of PIN protein abundance, homozygous ProPIN1::PIN1:GFP and ProPIN3::PIN3:GFP [43] or, for mapping of auxin maxima, ProDR5::GFP plants [48] were crossed with the XVE-bZIP11.4 line, respectively. Concerning gene expression analyses and root morphology assays, surface-sterilized and stratified seeds were cultivated on ¼ MS [61] agar plates without sugars under long day growth conditions (16 h light / 8 h dark) at 21°C and a relative humidity of 60%. Gene expression was analysed in 2-weeks old plants that were treated with 7 μM Est (Sigma-Aldrich Chemie GmbH, Munich, Germany) dissolved in phosphate buffered saline (PBS) for up to 24 h. For root morphology assays, plants were grown for 2 weeks on ¼ MS agar plates in vertical position before they were transferred to media supplemented with or without 0.25 μM NAA and/or 10 μM Est and cultivated for another week. ProDR5::GFP expression in aseptically grown XVE-bZIP lines was monitored after 24 h of Est- or solvent-treatment, respectively. Starvation assays applying DCMU were performed in liquid medium. Seeds of the XVE-bZIP2/11/44 knock-down line were incubated in 1 ml ¼ MS medium without sugars but -/+ 10 μM of Est in a 24 well plate for 3 days in the dark at 20°C in a plant growth incubator. After 3 more days, DCMU (10 μM) and/or glucose (1–3%) was/were added to the medium and plants were transferred to long day growth conditions. Root growth was determined 3 days after treatment.

### Chromatin immunoprecipitation

ChIP was described previously [21, 62] and was performed with minor modifications. In detail, 4 g of root material from aseptically grown XVE-bZIP11 plants was harvested at the middle of day after 8 h of Est treatment (10 μM) and incubated with cross-linking buffer (50 mM KH_2_PO_4_/K_2_HPO_4_ buffer (pH 5.8), 1% (v/v) formaldehyde) for 30 min under vacuum. Afterwards samples were incubated in glycine buffer (50 mM KH_2_PO_4_/K_2_HPO_4_ buffer (pH 5.8), 0.3 M glycine) for 15 min under vacuum and washed with ice-cold water. Samples were frozen in N_2_ and grinded. Nuclei extraction was performed at 4°C. Therefore, root material was resuspended in 24 ml ice-cold extraction buffer (1 M hexylenglycol, 50 mM PIPES-KOH (pH 7.2), 10 mM MgCl_2_, 5 mM ß-mercaptoethanol, one tablet per 10ml complete protease inhibitor cocktail tablets, Roche) and was cleared by filtrating it through two layers of miracloth. Afterwards 1 ml of 25% Triton X-100 was added dropwise to the extract. After incubation for 15 min, nuclei were isolated by density-gradient centrifugation using a 35% percoll cushion. The nuclei pellet was resuspended in sonication buffer (10 mM Tris-HCl (pH 7.4), 1 mM EDTA (pH 8.0), 0.25% SDS and protease inhibitor) prior to sonification for 20 times 30 s. Chromatin was cleared by centrifugation for 15 min at 11,000 g and 4°C and stored at -80°C. For each IP 10 μg chromatin and 3 μg ChIP grade a-HA antibody (ab9110) (Abcam, Cambridge, UK) were used. 70 μl of protein G-coated magnetic beads (Invitrogen, Karlsruhe, Germany) dissolved in ice-cold extraction buffer, supplemented with protease inhibitor (Roche, Mannheim, Germany) were applied to each sample. Antibody—antigen binding was achieved during a 4 h incubation step at 4°C and slow rotation on an Intelli-Mixer. Beads were washed five times with washing buffer supplemented with protease inhibitor, prior to elution of protein-DNA complexes using elution buffer (0.1 M glycine (pH 2.5), 500 mM NaCl, 0.05% (v/v) Tween-20). Precipitated DNA was quantified by q-RT-PCR using the oligonucleotide primers summarized in S2 Table. Data were normalized to DNA input, which was quantified by employing *ACTIN8* (At1g49240) specific primers. Presented mean and SEM values were calculated from two independent ChIP experiments that were performed on each of two independently prepared chromatin samples from WT or XVE-bZIP11 plants, respectively.

### Quantification of auxin-related root responses

To determine root morphology parameters, high resolution images of 40 individual plants per treatment were taken using the Camag reprostar 3 documentation system with a Canon G5 camera (CAMAG AG & Co. GmbH, Berlin, Germany). From these pictures, the root parameters of the differently treated plants were monitored. These are: the total primary root length before and after one week of treatment, the presence or absence of macroscopically visible root hairs and the abundance of roots with obvious agravitropic root growth. Root growth was considered to be agravitropic if roots showed at least one growth re-orientation of more than ~ 45° after inducer treatment. The root length was measured using the Image J 1.43u software (available at http://rsb.info.nih.gov/ij) whereas numbers of roots with/without root hairs and roots with agravitropic growth were determined by counting.

### Confocal laser scanning and bright-field microscopy

To determine GFP expression within the root, 200-fold enlarged bright-field and fluorescence images of individual root tips of ProPIN::PIN:GFP or ProDR5::GFP reporter lines were taken using the Leica SP8 confocal microscope. Fluorescence intensities were quantified as relative fluorescence intensity units using the Leica AF lite application suite 2.0.0. In order to quantify the root meristem size, 400-fold enlarged bright-field images of XVE-bZIP44 or XVE-bZIP11 plants were taken and analysed as previously described [36]. In detail, we focused on the QC cells and counted the expanding file of cortex cells originating from the QC up to the first strongly elongated cortex cell (at least twice in size) in the elongation zone. In the representative pictures in Fig 6A and 6B and S7A and S7B Fig we focused on the proposed boundary between meristem and elongation zone.

### Mass spectrometry analysis of auxin

Free IAA was measured using liquid chromatography-tandem mass spectrometry (LC-MS/MS) as described previously [63]. Briefly, 25–50 mg (fresh-weight) of *Arabidopsis* seedlings was extracted in sodium-phosphate buffer (pH 7). To each extract, 10 pmol of [^13^C_6_] IAA was added as internal standard to check recovery during purification and to validate the quantification. Samples were purified using a combination of a reversed-phase and anion-exchange chromatography and were analyzed by the LC-MS/MS system consisting of an ACQUITY UPLC System (Waters) and Xevo TQ MS (Waters). Samples were dissolved in 15% acetonitrile, injected onto the ACQUITY UPLC BEH C18 column (100 x 2.1 mm, 1.7 μm; Waters) and eluted with a linear gradient (0–3 min, 15% B; 3–10 min, 20% B; 10–20 min, 30% B; flow-rate of 0.25 ml min^-1^) of 7.5 mM formic acid (A) and acetonitrile (B). Quantification was obtained using a multiple reaction monitoring (MRM) mode of [M-H]^+^ and the appropriate product ion. The limits of detection (signal to noise ratio 1:3) for all analytes ranged from 5 to 10 fmol. The linear range was at least over the five orders of magnitude with a correlation coefficient of 0.9991–0.9997. For each genotype, at least five independent replicates were performed.

### Translating Ribosome Affinity Purification (TRAP)

TRAP method has been performed as described before [47]. In brief, 1.5 g of plant material were frozen in liquid nitrogen and homogenized to a fine powder using a mortar. The material was resuspended in twice the volume of ice-cold polysome extraction buffer (200 mM Tris-HCl, pH 9.0, 200 mM KCl, 25 mM EGTA, 36 mM MgCl_2_, 1% (v/v) octylphenyl-polyethylene glycol (Igepal CA-630), 1% (v/v) polyoxyethylene lauryl ether (Brig 35), 1% (v/v) Triton X-100, 1% (v/v) Tween-20, 1% (v/v) polyoxyethylene tridecyl ether, 1% (v/v) sodium deoxycholate, 1 mM dithiothreitol (DTT), 50 μg/ml cycloheximide, 50 μg/ml chloramphenicol) and incubated for 10 min on ice. The samples were centrifuged for 10 min at 4°C and 16.000 g. The obtained extract was again cleared by additional centrifugation (10 min at 4°C and 16.000 g) and filtration through two layers of miracloth. To precipitate FLAG-tagged ribosomes, 450 μl of magnetic beads coated with anti-FLAG antibodies (Sigma, Germany) were added to the extract. The mixture was incubated on an intellimixer for 3 hours at 4°C before the beads were collected on the side of the reaction tube using a magnet. The pellet was washed at least 5 times with 2 ml washing buffer (200 mM Tris-HCl, pH 9.0, 200 mM KCl, 25 mM EGTA, 36 mM MgCl_2_, 5 mM DTT, 50 μg/ml cycloheximide, 50 μg/ml chloramphenicol) before the ribosome-RNA complexes were eluted using anti-FLAG peptide (Sigma, Germany). The supernatant was collected and used to clean up the bound RNA using the RNEasy Micro Kit (Qiagen, Germany) following the manufacturer’s protocol. Isolated RNA was used for cDNA synthesis using oligo dT and random nonamer primers. To quantify *bZIP11* transcripts, 4 μl of cDNA were used for subsequent q-RT-PCR analysis employing *bZIP11* specific primers that are given in S2 Table.

### Molecular biological techniques

Standard DNA techniques have been described, previously [64]. DNA sequence analyses were performed by LGC Genomics (Berlin, Germany). Western analysis was performed making use of a polyclonal α-HA antibody from rabbit (Santa Cruz, Santa Cruz, CA, USA) and an anti-rabbit IgG conjugated with a horseradish peroxidase (GE Healthcare, Freiburg, Germany). q-RT-PCR has been performed with SYBR green as described, previously [10]. Data were obtained from 3 replicates of at least 2 individual plant pools and were normalized to *UBQ5* (At3g62250) transcript abundance. All q-RT-PCR oligonucleotide primers used are summarized in S2 Table.

### Statistics

Figures and statistical tests were performed applying the OriginPro 8.1G software. Significant differences between multiple constructs and treatments were determined using the One-way ANOVA test followed by a Tukey post-hoc test (p < 0.05) and are visualized by different letters. Significant differences between only two datasets were defined making use of the Student’s *t*-Test and labeled with asterisks (* p < 0.05; ** p < 0.01; *** p < 0.001).

## Supporting information

S1 Fig Molecular characterization of estradiol-inducible bZIP amiRNA plants. Transcript abundance of highly-related group S1 *bZIP* genes (*bZIP1*, *bZIP2*, *bZIP11*, *bZIP44* and *bZIP53*) was analysed by q-RT-PCR in XVE-ami2/11/44 (line #4) and WT plants in presence of 10 μM Est. Given are mean expression levels of *bZIP* genes (+/- SEM) from 3 biological replicates relative to *UBQ5* transcription and normalized to WT (set to 1). Significant differences between WT and XVE-ami2/11/44 plants for each gene were determined by Student’s *t*-Test and are marked by asterisks (* p ˂ 0.05; *** p ˂ 0.001).
(TIFF)

S2 Fig bZIP11-related TFs and IAA3/SHY2 control root growth in response to energy deprivation. **A**) Root increment of WT and transgenic XVE-amiRNA-bZIP2/11/44 (line 2) was determined after cultivating 1-week old plants for 3 more days in the presence of 10 μM Est, the photosynthetic inhibitor DCMU (10 μM) and increasing concentrations of glucose (0–3%). Presented is the mean root increment from 20 individual plants (+/-SEM), which is relative to the total primary root length (in %). Significant differences between genotypes for each individual treatment were determined by Student’s *t*-test and are labelled with asterisks (* p ˂ 0.05). Supplementary data referring to results shown in (**B**) Fig 2A or (**C**) Fig 2B. Given are representative pictures of root growth of WT (Col-0), (**B**) two independent XVE-ami2/11/44 lines (line #2 and #4) or (**C**) the *iaa3* loss-of-function mutant (*shy2-24* in Col-0 background) that was analysed in response to extended darkness. Differently coloured bars represent root length at the beginning of the day (yellow bars), the beginning of the extended night period (red bars) or after 24 hours of extended darkness (black bars). Scale bar: 1 cm.
(TIFF)

S3 Fig bZIP11-related TFs control auxin-related root phenotypes. **A**) Est-dependent expression of HA-tagged bZIP proteins was analysed in indicated XVE-lines by immuno-detection using an α-HA-tag antibody (upper panel). An unspecific protein band serves as loading control (lower panel). **B and C**) Auxin-related root growth parameters such as (**B**) primary root increment and (**C**) root gravitropism were analysed in WT, individual Est-inducible bZIP overexpression (XVE-bZIP2, line 2; XVE-bZIP44, line 3 and line 9) and knock-down (XVE-ami2/11/44, line 2) lines. Prior to analysis, plants were cultivated for 2 weeks on ½ MS plates without sugars under long day regime and then transferred for another week on inductive medium supplemented with 10 μM Est (red bars), exogenous auxin (0.25 μM NAA, blue bars), a combined Est and NAA treatment (black bars) or DMSO as solvent control (white bars). Presented is (**B**) the mean percentage of root increment relative to root length before treatment or (**C**) mean percentage of plants showing agravitropic roots growth (+/- SEM) determined from 40 individual plants per treatment and genotype. Statistically significant differences between the treatments for each genotype were determined by one-way ANOVA and Tukey post-hoc test and are labelled with different letters. **D and E**) 2-weeks old XVE-bZIP11 (line 4) plants were cultivated for 7 days on MS medium supplemented with (+/-) varying concentrations of NAA (0.001 to 1 μM) and/or Est (10 μM). Auxin-related root growth phenotypes were quantified with respect to (**D**) the increment of root length, and (**E**) agravitropic root growth. Mock (grey), Est (red), NAA (blue) and combined Est/NAA (black) treatments are visualised by differently coloured lines. Presented are mean values (+/- SEM) from 50 plants per treatment. Significant differences within a specific treatment were determined by one-way ANOVA and Tukey post-hoc test and denoted with different letters. Asterisks mark significant differences between two selected treatments, which were determined by Student’s *t*-Test (* p < 0.05; ** p < 0.01; *** p < 0.001).
(TIFF)

S4 Fig Energy deprivation as well as bZIP11 expression reduces root-tip-localised auxin and PIN levels. **A)** Root-tip auxin responsiveness and PIN3 abundance was analysed in response to extended night cultivation in respective ProPIN3::PIN3:GFP or ProDR5::GFP reporter lines using confocal microscopy. Given are mean GFP fluorescence values (+/- SEM) from at least 15 independent plants per genotype and time-point, which are relative to values obtained at the end of the normal night period (set to 100%). Significant differences compared to night samples were determined by Student’s *t*-Test and are labelled with asterisks (*** p < 0.001). **B**) PIN1 and PIN3 abundance was examined in transgenic XVE-bZIP11 plants that were crossed with ProPIN1::PIN1:GFP or ProPIN3::PIN3:GFP reporter lines. Presented are mean GFP fluorescence values (+/- SEM) from at least 5 independent plants per genotype and treatment (-/+ 10 μM Est for 16 h) and are relative to values obtained from uninduced plants (set to 100%). Significant differences between induced and un-induced samples were determined by Student’s *t*-Test and are labelled with asterisks (*** p < 0.001).
(TIFF)

S5 Fig Low-energy dependent *bZIP11* translation in the root stele. Energy dependent *bZIP11* translation rate in the root stele was determined by TRAP. Using transgenic lines expressing FLAG-tagged ribosomal RPL18 under control of a stele-specific promoter (ProSHR), ribosome-bound transcripts could be recovered by immunoprecipitation using FLAG antibodies coupled to magnetic beads. PCR amplification reveals a more than twofold increase in stele-specific translation of *bZIP11* transcripts under short-term (2 h) extended night conditions compared to that at the middle of the day. Presented are mean values (+/- SEM) from 3 independent experiments. Statistically significant differences between the treatments were determined by Student’s *t*-test and are labelled with asterisks (*** p ˂ 0.001).
(TIFF)

S6 Fig Root tip directed auxin transport is impaired by bZIP44. Root auxin levels were determined in WT and XVE-bZIP44 (line 9) by (**A and B**) introducing an auxin-sensitive ProDR5::GFP reporter construct or (**C**) direct measurements of free auxin (IAA) in distinct segments of the root. **A and B**) By this means it could be demonstrated that already 24 h after Est application, formation of the root tip-localized auxin maximum is strongly impaired. **A**) Given are representative pictures. The scale bar represents 150 μm. **B**) Auxin-driven GFP fluorescence in the WT and XVE-bZIP44 background was quantified from 40 individual plants per line and treatment in presence (grey bars) and absence (white bars) of Est (10 μM) and is expressed as mean fluorescence values (+/- SEM). Statistically significant differences between treatments were determined by Student’s *t*-test and are labelled with asterisks (*** p ˂ 0.001). **C**) Direct auxin measurements in the most distal root parts including root tip, the proximal root including hypocotyl and the shoot of WT and XVE-bZIP44 plants suggest an impairment of auxin transport from the shoot to the root tip. Mean auxin levels (+/- SEM) were determined after 6 h (grey bars) and 36 h (black bars) of Est treatment from at least 5 pools of plants per line and treatment and are given relative to WT levels (set to 1). Statistically significant differences between the genotypes for each time-point and root section were determined by Student’s *t*-test and are labelled with asterisks (* p ˂ 0.05;** p ˂ 0.01).
(TIFF)

S7 Fig bZIP44 represses meristematic root growth. RAM size was assessed by counting the file of cortex cells beginning from the QC to the first elongated cortex cell in the TZ. Therefore bright-field pictures of the root meristem of XVE-bZIP44 plants (line 9) after 24 h of (**A**) solvent or (**B**) Est-treatment were taken. Presented are representative images from 6 individual plants per treatment. Yellow boxes highlight cortex cells in the TZ and a red arrow indicates the border between root meristem and root elongation zone. The scale bar represents 20 μm. **C**) Mean meristem cell numbers (+/- SEM) from plants (n = 6) cultivated in the presence (grey bars) or absence (white bars) of Est. Statistically significant differences between treatments were determined by Student’s *t*-test and are labelled with asterisks (* p ˂ 0.05).
(TIFF)

S1 Table Auxin-related root hair growth is impaired by bZIP11-related TFs. Auxin application (0.25 μM NAA for 7 days) promotes local root hair formation distal to the root elongation zone. Est-induced bZIP2, -11 or -44 expression strongly impairs auxin-induced root hair growth. Given is the mean number of plants (+/- SEM) showing no macroscopically visible root hairs in the presence of auxin (NAA) or a combined NAA/Est treatment. Overall, roots of 40 individual plants per line and treatment were analysed. Statistically significant differences between treatments have been assigned by Student’s *t*-Test and are given as p-values (n.s. not significant).
(DOCX)

S2 Table List of oligonucleotides used in this study. (DOCX)
