# Supplementary figures and images for: The Arabidopsis bZIP11 transcription factor links low-energy signalling to auxin-mediated control of primary root growth
Source: PLoS Genet. 2017 Feb 3;13(2):e1006607. doi: 10.1371/journal.pgen.1006607 (PMC5315408; doi:10.1371/journal.pgen.1006607)

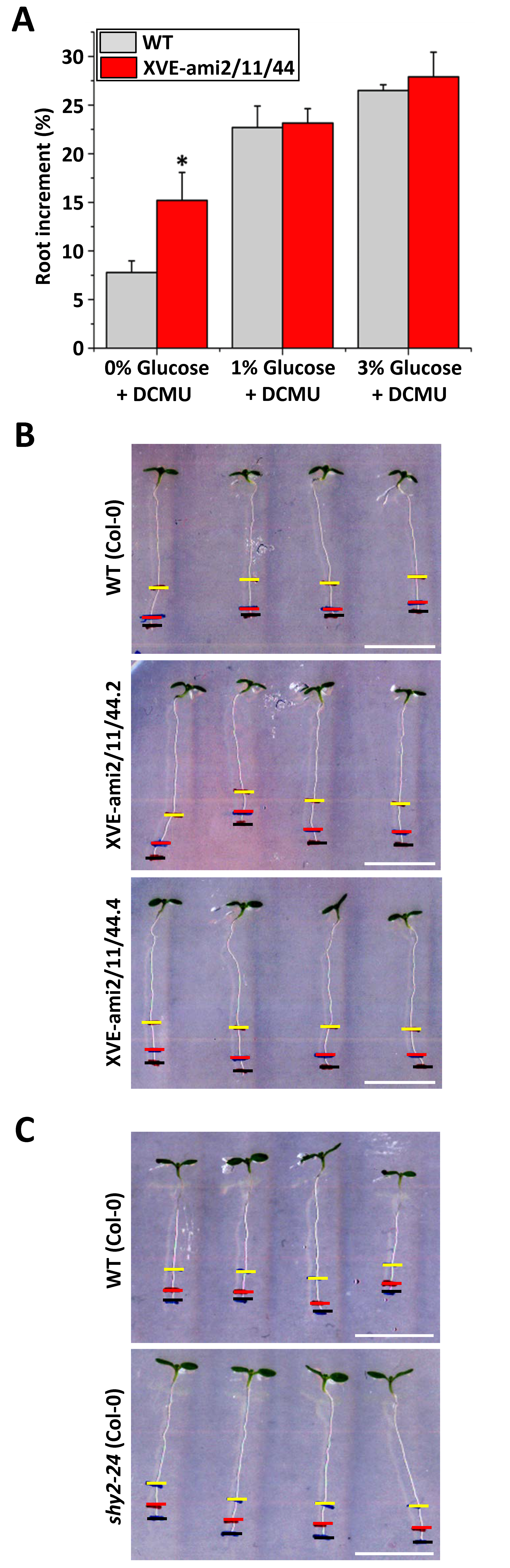

Supplement: S2 Fig — A) Root increment of WT and transgenic XVE-amiRNA-bZIP2/11/44 (line 2) was determined after cultivating 1-week old plants for 3 more days in the presence of 10 μM Est, the photosynthetic inhibitor DCMU (10 μM) and increasing concentrations of glucose (0–3%). Presented is the mean root increment from 20 individual plants (+/-SEM), which is relative to the total primary root length (in %). Significant differences between genotypes for each individual treatment were determined by Student’s t-test and are labelled with asterisks (* p ˂ 0.05). Supplementary data referring to results shown in (B) Fig 2A or (C) Fig 2B. Given are representative pictures of root growth of WT (Col-0), (B) two independent XVE-ami2/11/44 lines (line #2 and #4) or (C) the iaa3 loss-of-function mutant (shy2-24 in Col-0 background) that was analysed in response to extended darkness. Differently coloured bars represent root length at the beginning of the day (yellow bars), the beginning of the extended night period (red bars) or after 24 hours of extended darkness (black bars). Scale bar: 1 cm. (TIFF) [file pgen.1006607.s002.tiff]

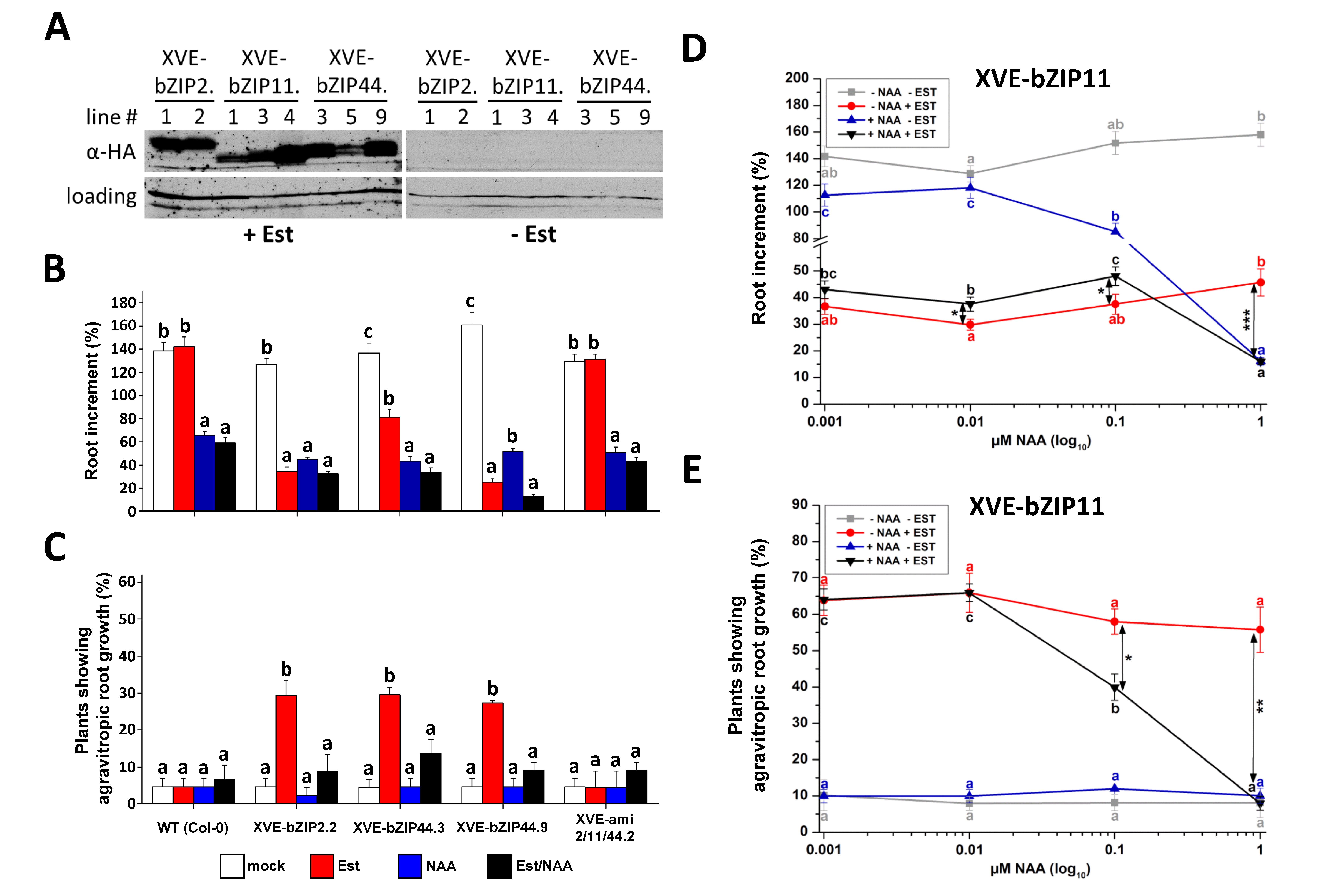

Supplement: S3 Fig — A) Est-dependent expression of HA-tagged bZIP proteins was analysed in indicated XVE-lines by immuno-detection using an α-HA-tag antibody (upper panel). An unspecific protein band serves as loading control (lower panel). B and C) Auxin-related root growth parameters such as (B) primary root increment and (C) root gravitropism were analysed in WT, individual Est-inducible bZIP overexpression (XVE-bZIP2, line 2; XVE-bZIP44, line 3 and line 9) and knock-down (XVE-ami2/11/44, line 2) lines. Prior to analysis, plants were cultivated for 2 weeks on ½ MS plates without sugars under long day regime and then transferred for another week on inductive medium supplemented with 10 μM Est (red bars), exogenous auxin (0.25 μM NAA, blue bars), a combined Est and NAA treatment (black bars) or DMSO as solvent control (white bars). Presented is (B) the mean percentage of root increment relative to root length before treatment or (C) mean percentage of plants showing agravitropic roots growth (+/- SEM) determined from 40 individual plants per treatment and genotype. Statistically significant differences between the treatments for each genotype were determined by one-way ANOVA and Tukey post-hoc test and are labelled with different letters. D and E) 2-weeks old XVE-bZIP11 (line 4) plants were cultivated for 7 days on MS medium supplemented with (+/-) varying concentrations of NAA (0.001 to 1 μM) and/or Est (10 μM). Auxin-related root growth phenotypes were quantified with respect to (D) the increment of root length, and (E) agravitropic root growth. Mock (grey), Est (red), NAA (blue) and combined Est/NAA (black) treatments are visualised by differently coloured lines. Presented are mean values (+/- SEM) from 50 plants per treatment. Significant differences within a specific treatment were determined by one-way ANOVA and Tukey post-hoc test and denoted with different letters. Asterisks mark significant differences between two selected treatments, which were determined by Student’s t-Test [file pgen.1006607.s003.tiff]

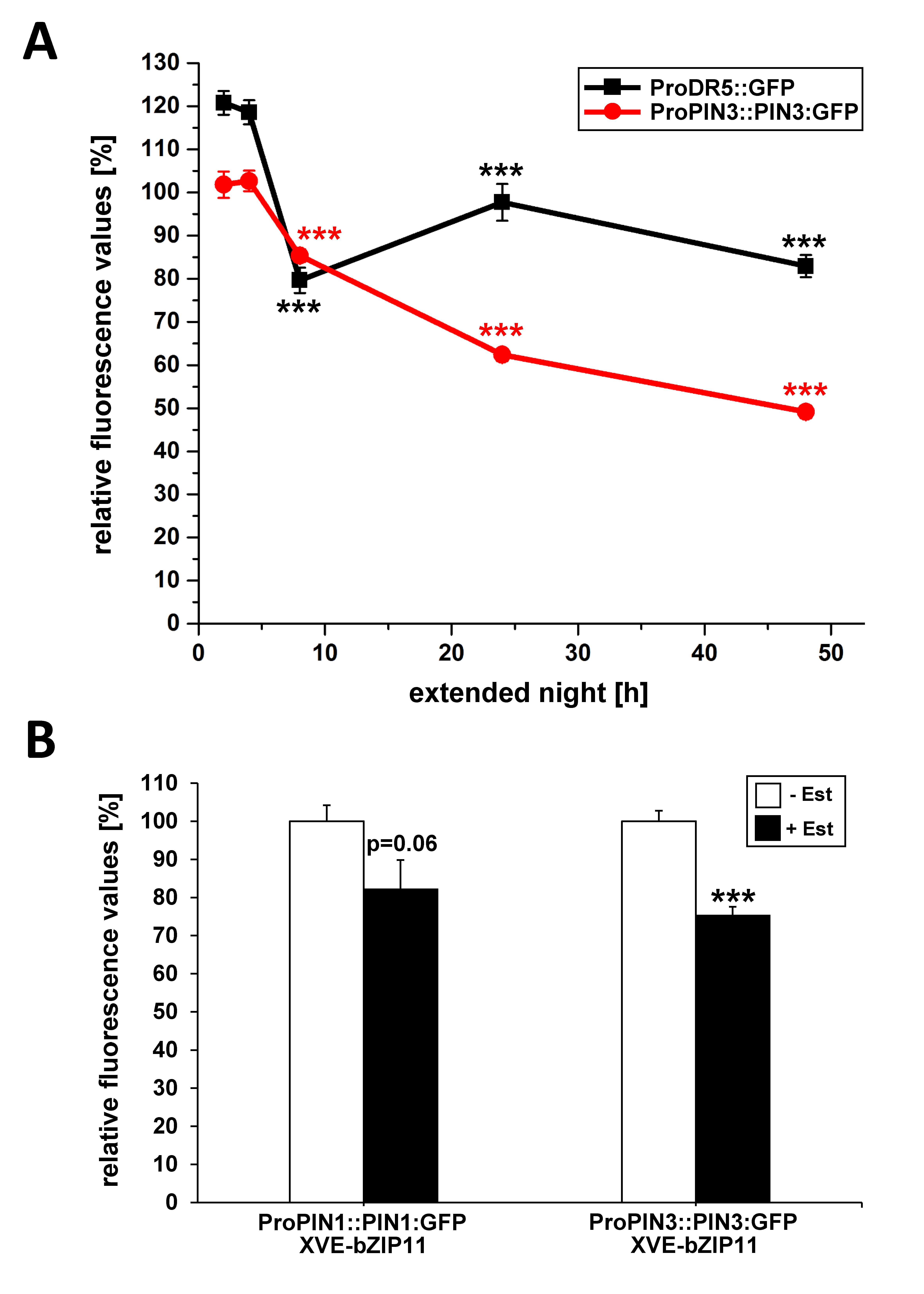

Supplement: S4 Fig — A) Root-tip auxin responsiveness and PIN3 abundance was analysed in response to extended night cultivation in respective ProPIN3::PIN3:GFP or ProDR5::GFP reporter lines using confocal microscopy. Given are mean GFP fluorescence values (+/- SEM) from at least 15 independent plants per genotype and time-point, which are relative to values obtained at the end of the normal night period (set to 100%). Significant differences compared to night samples were determined by Student’s t-Test and are labelled with asterisks (*** p < 0.001). B) PIN1 and PIN3 abundance was examined in transgenic XVE-bZIP11 plants that were crossed with ProPIN1::PIN1:GFP or ProPIN3::PIN3:GFP reporter lines. Presented are mean GFP fluorescence values (+/- SEM) from at least 5 independent plants per genotype and treatment (-/+ 10 μM Est for 16 h) and are relative to values obtained from uninduced plants (set to 100%). Significant differences between induced and un-induced samples were determined by Student’s t-Test and are labelled with asterisks (*** p < 0.001). (TIFF) [file pgen.1006607.s004.tiff]

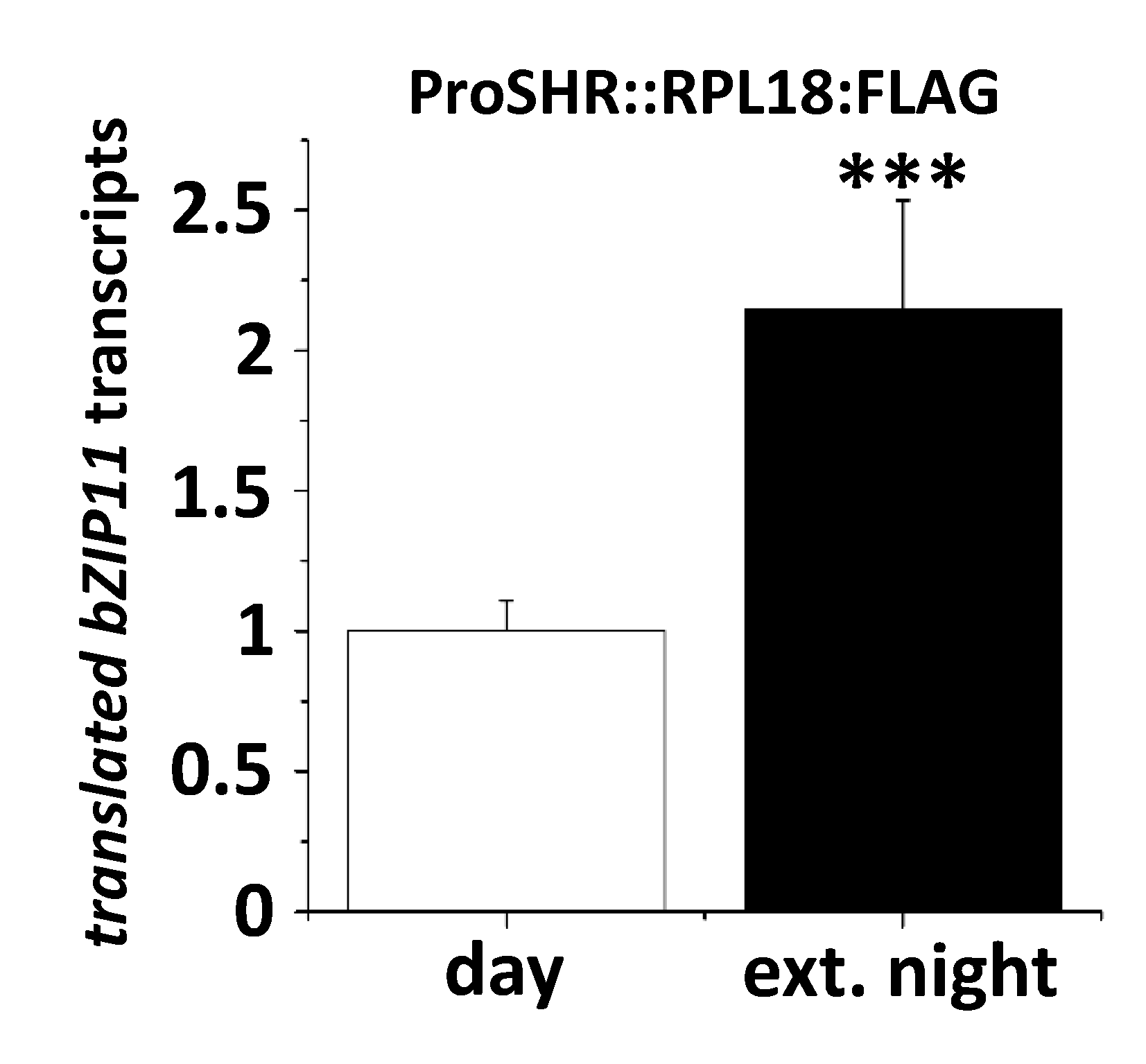

Supplement: S5 Fig — Energy dependent bZIP11 translation rate in the root stele was determined by TRAP. Using transgenic lines expressing FLAG-tagged ribosomal RPL18 under control of a stele-specific promoter (ProSHR), ribosome-bound transcripts could be recovered by immunoprecipitation using FLAG antibodies coupled to magnetic beads. PCR amplification reveals a more than twofold increase in stele-specific translation of bZIP11 transcripts under short-term (2 h) extended night conditions compared to that at the middle of the day. Presented are mean values (+/- SEM) from 3 independent experiments. Statistically significant differences between the treatments were determined by Student’s t-test and are labelled with asterisks (*** p ˂ 0.001). (TIFF) [file pgen.1006607.s005.tiff]

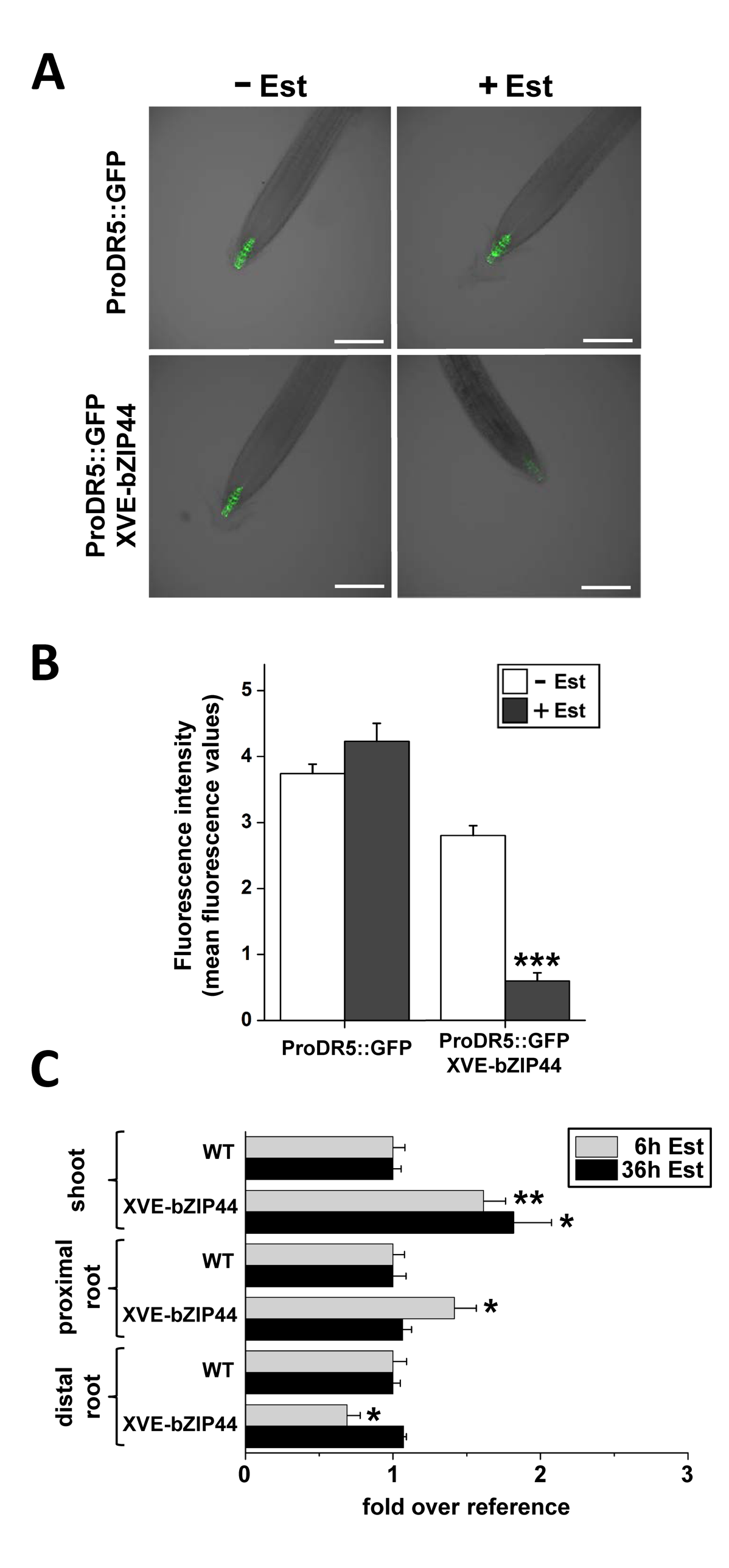

Supplement: S6 Fig — Root auxin levels were determined in WT and XVE-bZIP44 (line 9) by (A and B) introducing an auxin-sensitive ProDR5::GFP reporter construct or (C) direct measurements of free auxin (IAA) in distinct segments of the root. A and B) By this means it could be demonstrated that already 24 h after Est application, formation of the root tip-localized auxin maximum is strongly impaired. A) Given are representative pictures. The scale bar represents 150 μm. B) Auxin-driven GFP fluorescence in the WT and XVE-bZIP44 background was quantified from 40 individual plants per line and treatment in presence (grey bars) and absence (white bars) of Est (10 μM) and is expressed as mean fluorescence values (+/- SEM). Statistically significant differences between treatments were determined by Student’s t-test and are labelled with asterisks (*** p ˂ 0.001). C) Direct auxin measurements in the most distal root parts including root tip, the proximal root including hypocotyl and the shoot of WT and XVE-bZIP44 plants suggest an impairment of auxin transport from the shoot to the root tip. Mean auxin levels (+/- SEM) were determined after 6 h (grey bars) and 36 h (black bars) of Est treatment from at least 5 pools of plants per line and treatment and are given relative to WT levels (set to 1). Statistically significant differences between the genotypes for each time-point and root section were determined by Student’s t-test and are labelled with asterisks (* p ˂ 0.05;** p ˂ 0.01). (TIFF) [file pgen.1006607.s006.tiff]

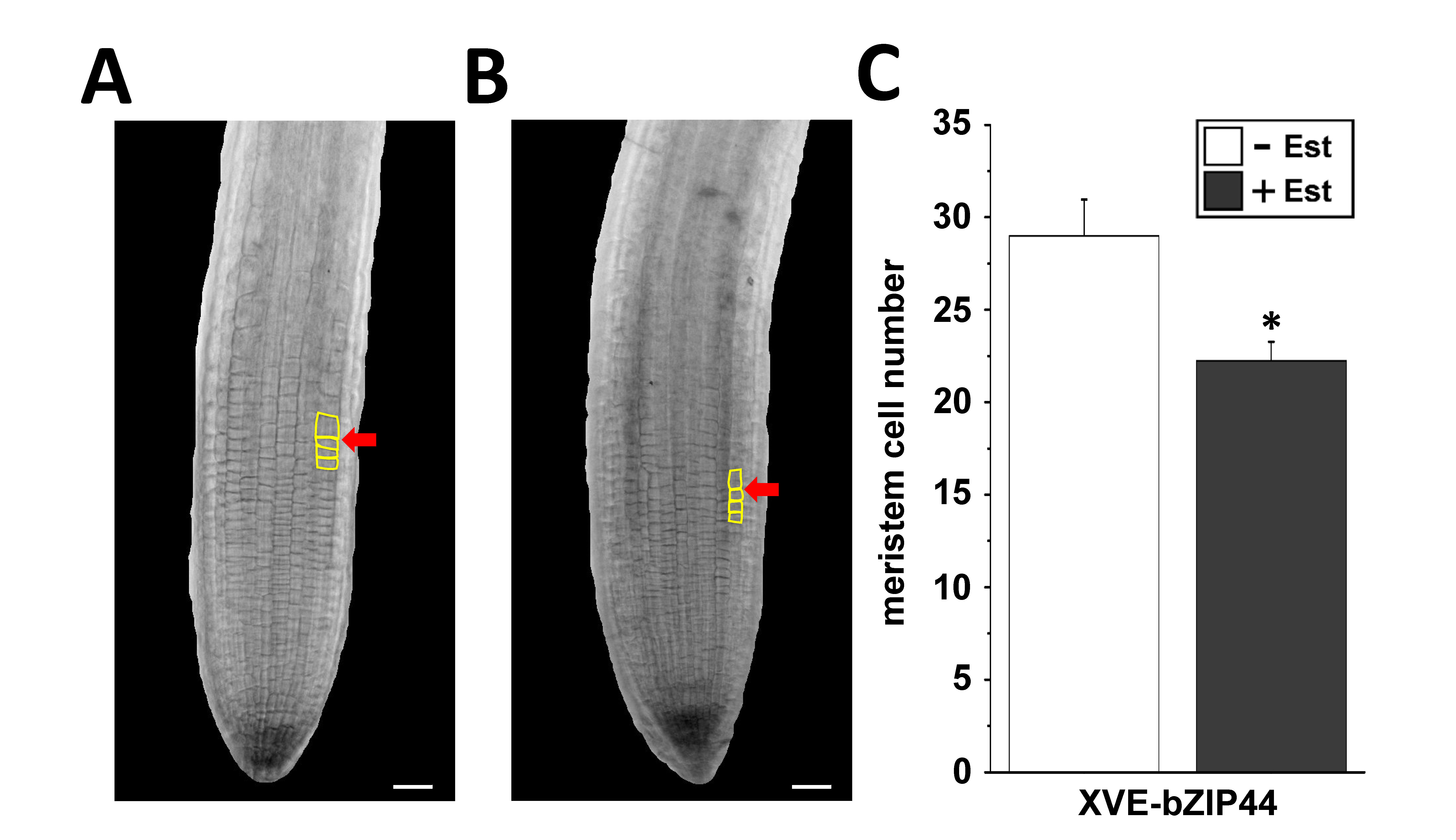

Supplement: S7 Fig — RAM size was assessed by counting the file of cortex cells beginning from the QC to the first elongated cortex cell in the TZ. Therefore bright-field pictures of the root meristem of XVE-bZIP44 plants (line 9) after 24 h of (A) solvent or (B) Est-treatment were taken. Presented are representative images from 6 individual plants per treatment. Yellow boxes highlight cortex cells in the TZ and a red arrow indicates the border between root meristem and root elongation zone. The scale bar represents 20 μm. C) Mean meristem cell numbers (+/- SEM) from plants (n = 6) cultivated in the presence (grey bars) or absence (white bars) of Est. Statistically significant differences between treatments were determined by Student’s t-test and are labelled with asterisks (* p ˂ 0.05). (TIFF) [file pgen.1006607.s007.tiff]
