# Supplementary material for: The Arabidopsis bZIP11 transcription factor links low-energy signalling to auxin-mediated control of primary root growth
Source: PLoS Genet. 2017 Feb 3;13(2):e1006607. doi: 10.1371/journal.pgen.1006607 (PMC5315408; doi:10.1371/journal.pgen.1006607)
Supplement: S1 Table — Auxin application (0.25 μM NAA for 7 days) promotes local root hair formation distal to the root elongation zone. Est-induced bZIP2, -11 or -44 expression strongly impairs auxin-induced root hair growth. Given is the mean number of plants (+/- SEM) showing no macroscopically visible root hairs in the presence of auxin (NAA) or a combined NAA/Est treatment. Overall, roots of 40 individual plants per line and treatment were analysed. Statistically significant differences between treatments have been assigned by Student’s t-Test and are given as p-values (n.s. not significant). (DOCX) [file pgen.1006607.s008.docx]

|  | **Plants without root hairs (%)** | |  |
| --- | --- | --- | --- |
| **Genotype** | **NAA** | **NAA + Est** | **p-value** |
| **WT (Col-0)** | 13.81 ± 4.27 | 18.10 ± 4.29 | 0.26 (n.s.) |
| **XVE-bZIP2.2** | 11.43 ± 2.40 | 93.17 ± 0.16 | 2.22E-06 |
| **XVE-bZIP11.3** | 15.87 ± 2.08 | 93.17 ± 0.16 | 1.59E-06 |
| **XVE-bZIP11.4** | 11.43 ± 2.40 | 95.56 ± 2.22 | 6.77E-06 |
| **XVE-bZIP44.3** | 27.30 ± 0.63 | 88.73 ± 2.06 | 4.54E-06 |
| **XVE-bZIP44.9** | 13.65 ± 3.86 | 97.78 ± 2.22 | 2.32E-05 |
| **XVE-ami2/11/44.2** | 29.52 ± 1.98 | 31.75 ± 1.59 | 0.22 (n.s.) |
