# Supplementary material for: The Arabidopsis bZIP11 transcription factor links low-energy signalling to auxin-mediated control of primary root growth
Source: PLoS Genet. 2017 Feb 3;13(2):e1006607. doi: 10.1371/journal.pgen.1006607 (PMC5315408; doi:10.1371/journal.pgen.1006607)
Supplement: S2 Table — (DOCX) [file pgen.1006607.s009.docx]

| **Primer** | **Sequence (5`-3`)** |
| --- | --- |
| ***bZIP1* qPCR for** | TGGCAAACGCAGAGAAGACAAGT |
| ***bZIP1* qPCR rev** | CGACCTCCTTGCAGATTCGCGG |
| ***bZIP2* qPCR for** | TGATCGGAAACTGATGACTCC |
| ***bZIP2* qPCR rev** | GAGCAGATTTGACCGTGAGC |
| ***bZIP11* qPCR for** | CGATTCAAACGTCGTCAGG |
| ***bZIP11* qPCR rev** | TCCGTTTACGTTTCCTCTGC |
| ***bZIP44* qPCR for** | CATCTACGTAAAGAAAACGCTCAG |
| ***bZIP44* qPCR rev** | CCGGTCTCCATACCGAATC |
| ***bZIP53* qPCR for** | TGGGGTCGTTGCAAATGCAAACAA |
| ***bZIP53* qPCR rev** | CCGTGGCGTACCTCGGATCATTAT |
| ***IAA3/SHY2* qPCR for** | AAAGGCTCAGATTGTTGGATGGC |
| ***IAA3/SHY2* qPCR rev** | TGACCCTCATGCTCAGATTCATTC |
| ***PIN1* qPCR for** | GGCATGGCTATGTTCAGTCTTGGG |
| ***PIN1* qPCR rev** | ACGGCAGGTCCAACGACAAATC |
| ***PIN3* qPCR for** | AAGGCGGAAGATCTGACCAAGG |
| ***PIN3* qPCR rev** | TGCTGGATGAGCTACAGCTTTG |
| ***UBQ5* qPCR for** | GACGCTTCATCTCGTCC |
| ***UBQ5* qPCR rev** | GTAAACGTAGGTGAGTCCA |
| **ChIP Pro*IAA3* -1800 for** | TGTGGGACCTGCATCTTCTG |
| **ChIP Pro*IAA3* -1800 rev** | ATTTGCTTGCCCTACAGAGC |
| **ChIP Pro*IAA3* -600 for** | ATACAACCAATGCCACGCTG |
| **ChIP Pro*IAA3* -600 rev** | ATCACCACACACACACACAC |
| **ChIP *IAA3* gene for** | GCCAAAGGTTTAGGCTGTGG |
| **ChIP *IAA3* gene rev** | AGATCTTGCCCGAGAAACCC |
| **ChIP *IAA3* 3`UTR for** | ACCTCTCTGCTTTCATGGCA |
| **ChIP *IAA3* 3`UTR rev** | CTCCCGCCATGTCTGAGAAA |
| **ChIP Pro*ACTIN7* for** | CGTTTCGCTTTCCTTAGTGTTAGCT |
| **ChIP Pro*ACTIN7* rev** | AGCGAACGGATCTAGAGACTCACCT |
| **ChIP *ACTIN8* for** | GGTTTTCCCCAGTGTTGTTG |
| **ChIP *ACTIN8* rev** | CTCCATGTCATCCCAGTTGC |
